# Pregestational Diabetes and Adverse Pregnancy Results: A Mendelian Randomization Study

**DOI:** 10.34172/aim.33461

**Published:** 2025-02-01

**Authors:** Sedigheh Hantoushzadeh, Majid Zaki-Dizaji, Danial Habibi, Leyla Sahebi, Amir Hesam Saeidian, Mohadese Dashtkoohi, Mostafa Saeedinia, Hanifeh Mirtavoos-Mahyar, Zohreh Heidary

**Affiliations:** ^1^Vali-E-Asr Reproductive Health Research Center, Family Health Research Institute, Tehran University of Medical Sciences, Tehran, Iran; ^2^Legal Medicine Research Center, Legal Medicine Organization, Tehran, Iran; ^3^Department of Epidemiology and Biostatistics, School of Public Health, Babol University of Medical Sciences, Babol, Iran; ^4^Maternal, Fetal and Neonatal Research Center, Family Health Research Institute, Tehran University of Medical Sciences, Tehran, Iran; ^5^Department of Surgery, Rasool-E Akram Hospital, School of Medicine, Iran University of Medical Sciences, Tehran, Iran; ^6^Shahid Beheshti University of Medical Sciences, Tehran, Iran; ^7^Lung Transplantation Research Center, National Research Institute of Tuberculosis and Lung Diseases, Shahid Beheshti University of Medical Sciences, Tehran, Iran

**Keywords:** Abortion, Diabetes mellitus, Maternal diabetes mellitus, Pregnancy outcome, Preterm birth, Spontaneous, Stillbirth

## Abstract

**Background:**

Hyperglycemia in pregnancy is believed to be associated with negative pregnancy outcomes. However, establishing a causal connection between diabetes mellitus (DM) and adverse pregnancy results is challenging due to the limitations inherent in traditional observational studies.

**Methods:**

Our study used a two-sample Mendelian randomization (MR) technique to examine the possible influence of pregestational diabetes mellitus (PGDM) on adverse pregnancy outcomes. Summary-level data were obtained from genome-wide association studies (GWAS) of European ancestry and FinnGen biobank. The primary analysis employed the random-effects multiplicative inverse variance weighted (IVW) technique to appraise causal relationships between PGDM and adverse outcomes. Heterogeneity and pleiotropy were assessed using Cochran’s Q statistic, Rucker’s Q statistic, and the I^2^ statistic. Sensitivity analyses were conducted using MR-Egger and weighted median methods. Additionally, outlier detection techniques, including MR-PRESSO and RadialMR, were applied.

**Results:**

The results from the IVW method indicated no significant causal association between PGDM and stillbirth (SB) (OR (SE)=0.99 (0.001); *P* value=0.992), miscarriage (MIS) (OR (SE)=0.97 (0.016); *P* value=0.125), and preterm birth (PTB) (OR (SE)=1.072 (0.028); *P* value=0.014). Pleiotropy and heterogeneity tests revealed no evidence of pleiotropy for SB, MIS, and PTB (MR–Egger intercept *P* value=0.296, 0.525, and 0.532, respectively), with no observed heterogeneity for SB, MIS, and PTB (Q- *P* values of IVW were 0.929, 0.999, and 0.069, and MR–Egger were 0.931, 0.999, and 0.065, respectively).

**Conclusion:**

Our findings indicate that there is no direct causal link between PGDM and the likelihood of MIS, SB, and PTB.

## Introduction

 The overall prevalence of diabetes mellitus (DM) has elevated due to population aging, economic development, and a shift towards sedentary lifestyles over the past few decades. It is estimated that by 2045, DM will affect approximately 451 million individuals aged 18 years and older worldwide, with type 2 diabetes (T2DM) and its associated conditions being the primary components of this epidemic.^1-4^ Moreover, hyperglycemia during pregnancy is a global health issue affecting a significant number of women and is and is connected to a range of adverse pregnancy results.^5^

 Preterm birth (PTB) and stillbirth (SB) have a significant impact on around 19 million women globally each year, with reports from the World Health Organization indicating the rising prevalence of these outcomes.^6^ Miscarriage (MIS) affects approximately 23 million women annually, equivalent to an average of 44 cases per minute.^7^ These adverse pregnancy results are associated with high rates of morbidity and mortality.^8,9^ The rate of SB among pregnant women with diabetes is approximately 20 per 1000 births,^10^ and a recent study found a PTB prevalence of 17.72% among mothers with gestational diabetes mellitus (GDM).^11^ Both pregestational diabetes mellitus (PGDM) and GDM are linked to higher chances of adverse maternal and neonatal outcomes, particularly in cases of PGDM.^5,12^ The mechanisms connecting PGDM with MIS, SB, and PB are not fully understood, but there is evidence suggesting that high blood sugar and insulin levels may contribute to fetal hypoxia and acidosis in the womb, leading to SB and other negative pregnancy outcomes.^10,13^

 Establishing a causal relationship between PGDM and adverse pregnancy outcomes remains challenging due to confounding factors and limitations of traditional observational studies. To overcome these limitations, the Mendelian randomization (MR) approach offers a promising avenue and evaluates the causal effects of maternal PGDM on these adverse pregnancy outcomes.^14^ By leveraging the random assortment of genetic variants, MR provides a robust framework to assess causality while minimizing biases inherent in traditional observational studies.^15^

 In this study, we used an MR approach to investigate the causal relationship between PGDM and SB, MIS, and PTB.

## Materials and Methods

###  Study Design

 The current study adheres to the Strengthening the Reporting of Observational Studies in Epidemiology-MR statement (The STROBE-MR Statement).^16^

**Figure 1:**
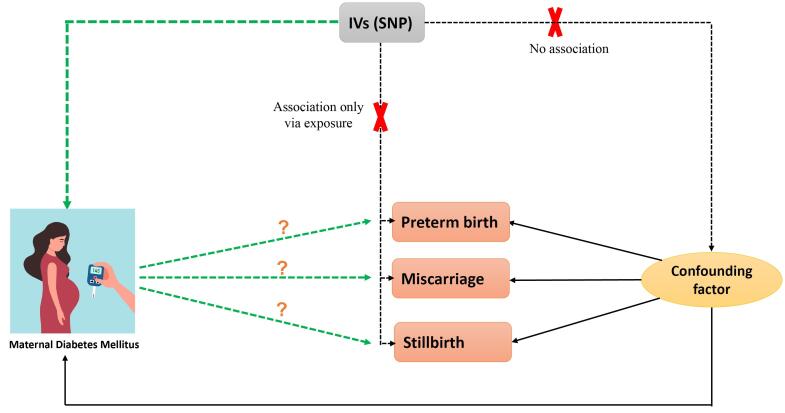
Flow Chart of the Two-Sample Mendelian Randomization Study Design

 Our study applied publicly available summary-level data from genome-wide association studies (GWAS). Specifically, data were obtained from the GWAS catalog and OpenWAS (https://www.ebi.ac.uk/gwas/ and https://gwas.mrcieu.ac.uk/) and consisted of genetic associations from independent GWAS datasets with matching ancestral backgrounds to mitigate confounding factors (Figure 1). The analysis using summarized data is outlined in Table 1.

**Table 1:** Detailed Data Description.

**Name**	**GWAD ID**	**Sample Size**	**Role**
Stillbirth	Ukb-a-321	461 880 (case = 56 172, control = 122 302)	Outcome
Miscarriage	Finngen (R9-015_ABORT_SPONTAN)	463 010 (case = 16 906, control = 149 622)	Outcome
Preterm	Finngen (finn-b-O15_PRETERM)	104 106 (case = 5480, control = 98 626)	Outcome
Diabetes mellitus	GCST90132184	361 194 (case = 180 834, control = 1 159 055)	Exposure

###  Genetic Instrument Selection

**Figure 2:**
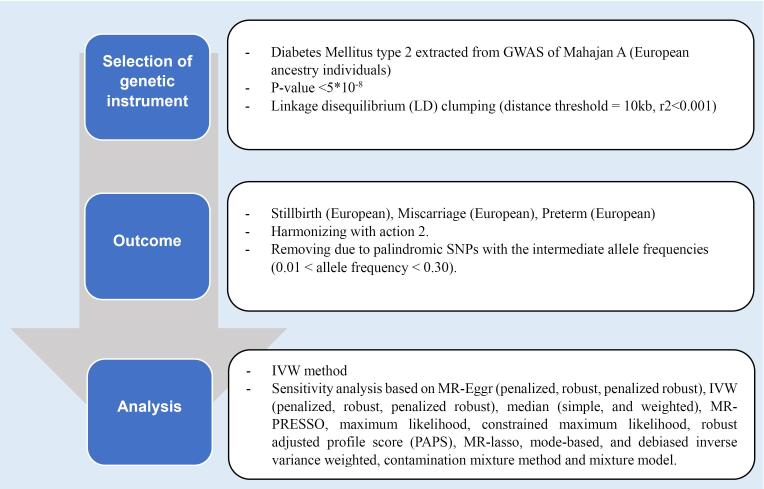
Mendelian Randomization Design

 This step ensured that single nucleotide polymorphisms (SNPs) met the criteria linking them to DM and were independent of factors influencing adverse pregnancy outcomes (Figure 2).

 A total of 187 SNPs strongly associated with DM were identified as instrumental variables based on stringent statistical thresholds (P < 5 × 10^-8^) and linkage disequilibrium parameters. Further refinement excluded SNPs with potential horizontal polymorphic effects. The validity of instrumental variables was assessed using F-statistics, with values greater than 10 indicating compliance with the first assumption.

###  Two-Sample Mendelian Randomization Analysis

 The random-effects multiplicative inverse variance weighted (IVW) method was primarily employed to assess the relationship between DM and adverse pregnancy outcomes. Various statistical tests were utilized to assess heterogeneity and pleiotropy, including Cochran’s Q statistic, Rucker’s Q statistic, and the I^2^ statistic.

 Sensitivity analyses, such as MR-Egger and weighted median methods, were conducted to ensure the robustness of results against potential biases. Outlier detection techniques, including MR-PRESSO and RadialMR, were employed to identify and mitigate pleiotropic effects.

 Statistical analyses were conducted using the R software (version 4.0.3) with relevant packages and STATA (version 17). Results were presented as odds ratios (OR) with corresponding 95% confidence intervals (CIs), with associations possessing *P* values below 0.05 considered significant.

## Results

###  SNPs Selection 

 From 10 454 801 SNPs of DM, we earned 187 SNPs with a significant genome-wide threshold (P < 5 × 10^-8^) and clumping. Following the harmonization process, 186 and 180 SNPs remained for SB and PTB, respectively, but we attained 177 SNPs for MIS. Also, all SNPs for SB and PTB were kept on after removing the human leukocyte antigen region, and minor allele frequency was less than 0.01, but one SNP was removed for MIS.

 We conducted pleiotropy checks for the SNPs to detect potential confounders. After checking by PhenoScanner, 5 SNPs were omitted for SB (N = 181), and all SNPs remained for MIS and PTB (N = 176 and N = 180, respectively). In checking weaknesses in the instruments, all SNPs remained in the analysis.

###  Mendelian Randomization Analysis

 The results of the IVW method showed no significant causal relationship between DM with SB, MIS, and PTB ([OR = 0.99, 95% CI: -0.004, 0.004; *P* value = 0.992]; [OR = 0.97, 95% CI: -0.056, 0.007; *P* value = 0.125], [OR = 1.072, 95% CI: 0.014, 0.126; *P* value = 0.014], respectively). According to the pleiotropy and heterogeneity test, there was no pleiotropy for SB, MIS, and PTB (*P* value of MR–Egger intercept: 0.296, 0.525, and 0.532, respectively). Also, heterogeneity was not observed for SB, MIS, and PTB (Q-*P* values of IVW: 0.929, 0.999 and 0.069; and MR–Egger: 0.931, 0.999 and 0.065, respectively; I^2^_SB_ = 0%, 95% CI: 0, 19.2; I^2^_MIS_ = 0%, 95% CI: 0, 19.6; I^2^_PR_ = 14.2%, 95% CI: 0, 29.5, I^2^_GX for SB_ = 91.88%, I^2^_GX for MIS_ = 92.39%, I^2^_GX for PR_ = 92.68%). A step-by-step demonstration of all the results is provided in Supplementary file 1.

###  Sensitivity Analysis

**Figure 3:**
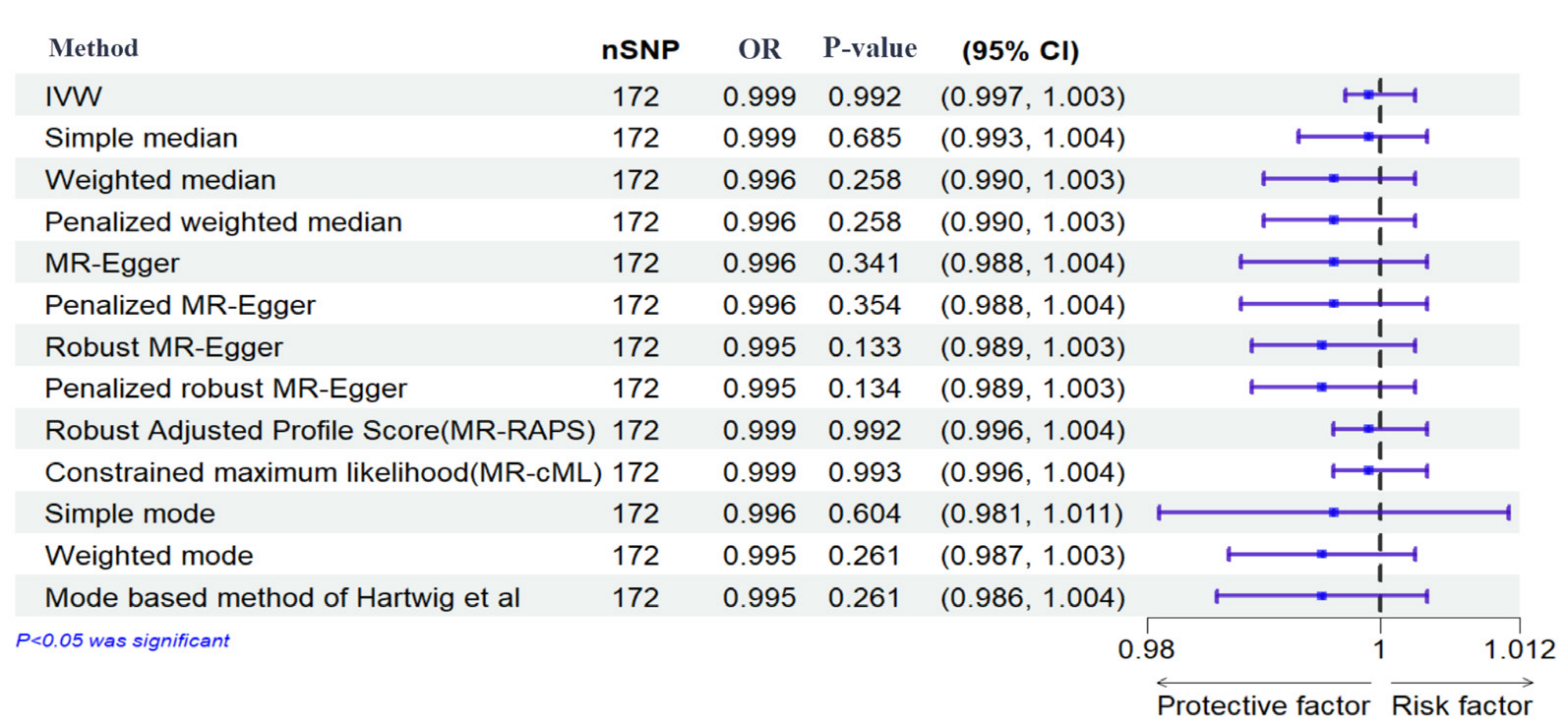
Effects of Genetically Determined Maternal Diabetes Mellitus and Stillbirth. Here, maternal diabetes mellitus and stillbirth are exposure and outcome, respectively. nSNPs: number of single-nucleotide polymorphisms

**Figure 4:**
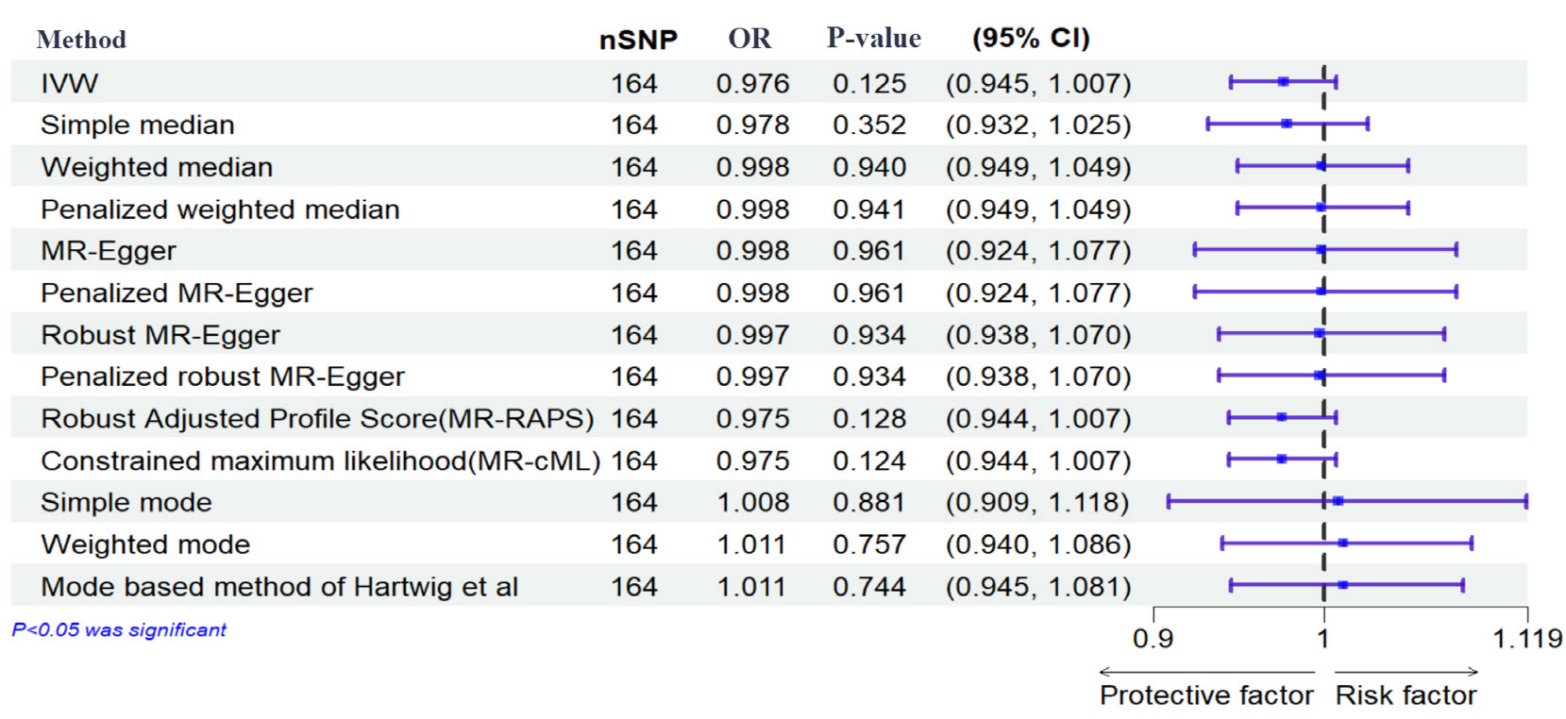
Effects of Genetically Determined Maternal Diabetes Mellitus and Miscarriage. Here, maternal diabetes mellitus and miscarriage are exposure and outcome, respectively. nSNPs: number of single-nucleotide polymorphisms

**Figure 5:**
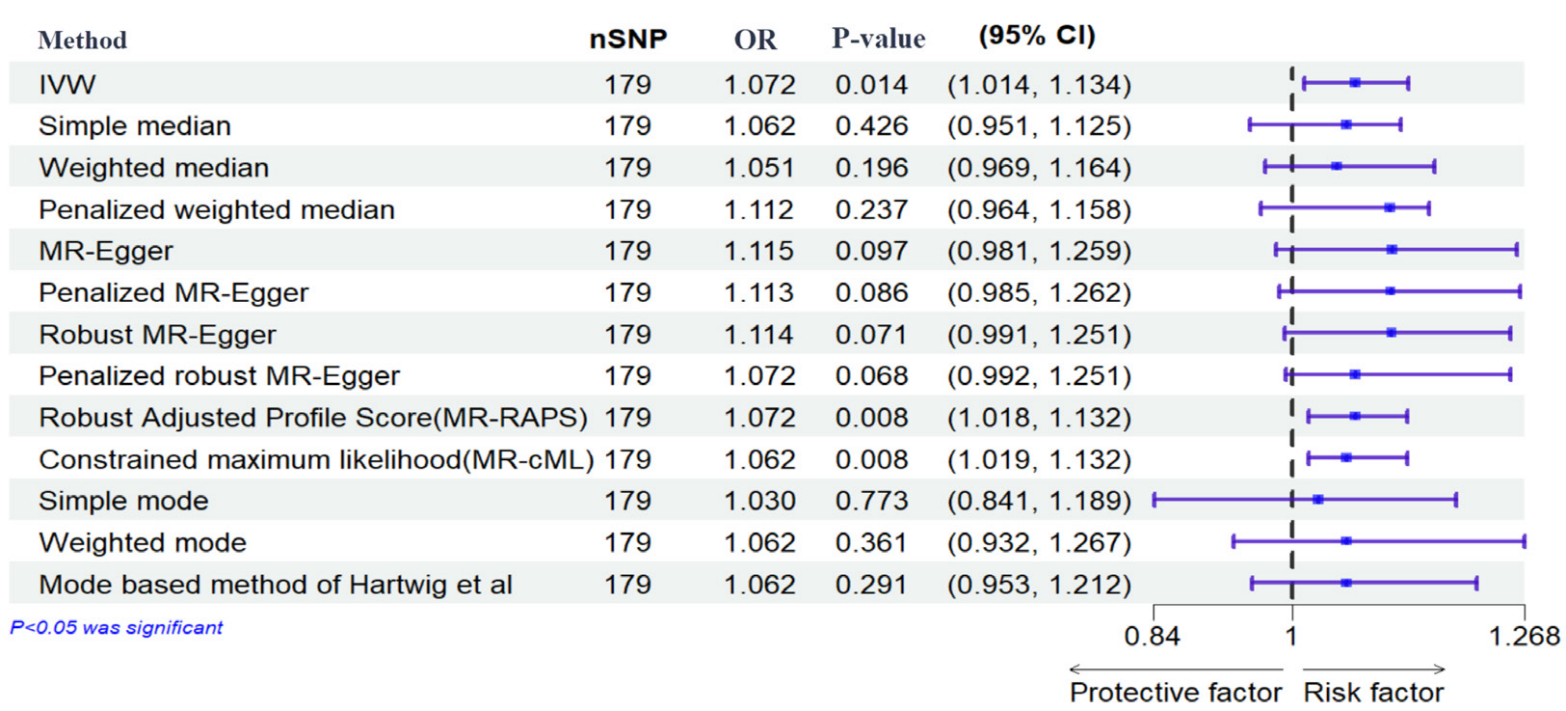
Effects of Genetically Determined Maternal Diabetes Mellitus and Preterm. Here, maternal diabetes mellitus and preterm are exposure and outcome, respectively. nSNPs: number of single-nucleotide polymorphisms

 We used the MR pleiotropy residual sum and outlier test (MR-PRESSO) methods, MR pleiotropy residual sum and outlier (Radial MR), Cook’s distance, and Studentized residuals to identify outliers or influential observations. Among them, Cook’s distance outperformed. Cook’s distance can be used to (1) indicate influential data points worth checking for validity; and (2) indicate regions of the design space where it would be good to obtain more data points. Based on this method, 9 SNPs were excluded in SB, and 8 SNPs were excluded for MIS (Supplementary files 2-4). Moreover, Leave-one-SNP-out analysis and plot were performed to assess the influence of potentially pleiotropic SNPs on the causal estimation. Funnel and forest plots were used to detect directional pleiotropy and visual association genetic association, respectively. These findings remained consistent in sensitivity analysis using different MR methods and sensitivity analyses (Figures 3-5, Supplementary file 1).

## Discussion

 Diabetes during pregnancy is a recognized risk factor for a spectrum of pregnancy complications, with associated elevations in maternal and fetal morbidity and mortality rates.^17,18^ Approximately 10% of cases of maternal diabetes are attributed to PGDM.^19^

 Analyzing a cohort of 10 734 mothers of European descent, to find a causal link between PGDM and pregnancy complications including MIS, SB, and PTB, we did not substantiate a significant causal link.

 Maternal diabetes, regardless of its etiology, is established as a risk factor for pregnancy complications. However, the severity of adverse outcomes is notably higher in cases of PGDM.^20-24^ Poorly managed PGDM, particularly during the critical first trimester, is correlated to elevated incidence of congenital anomalies, MIS, SB, and PTB.^25,26^

 An established association exists between diabetes and SB, with reported rates for PGDM exceeding 9% compared to 0.5% in GDM cases.^21^ The Scottish Morbidity Record underscored this disparity, revealing SB rates to be four to five times higher among women with type 1 and type 2 diabetes compared to their counterparts.^27^ Contributing factors to SB in diabetic pregnancies include hyperglycemia, obesity, prior cesarean delivery, fetal anomalies, and intrauterine growth restriction.^28^ A meta-analysis encompassing 70 studies by Syed et al^29^ demonstrated a substantial 10% reduction in SB rates through effective diabetes management and surveillance.

 PTB and MIS are also more prevalent in pregnancies with PGDM compared to GDM and non-diabetic pregnant women. Van Zyl and Levitt^21^ reported PTB rates of 68.8% for T1DM, 38.7% for T2DM, and 34.9% for GDM. Soliman et al^30^ reported significantly elevated PTB rates among women with PGDM (13.7%) and GDM (9%) relative to a control group (6.4%). Concerning HbA1c levels, it was found that the risk of MIS was 12.4% when HbA1c levels were at or below 9.3% in the first trimester, and 37.5% when HbA1c levels exceeded 14.4%.^31^

 Previous research has elucidated the genetic underpinnings of diabetes susceptibility, identifying distinct genetic profiles associated predominantly with T2DM and GDM, with certain overlapping features.^32,33^

 However, MR analyses offer a significant advantage by assessing causal relationships without the confounding influence present in observational studies.^14^ This methodological strength may explain the discrepancies between our MR findings and previous observational research, which often reported associations between PGDM and adverse pregnancy outcomes.

 Confounding factors, such as maternal obesity, can substantially impact the observed relationship. Maternal obesity, advanced age, and a sedentary lifestyle are key risk factors for diabetes.^34,35^ Obesity is a well-established risk factor for both PGDM and adverse pregnancy outcomes. The chronic inflammatory state associated with obesity can contribute to insulin resistance and pregnancy complications.^36^ Moreover, hyperglycemia-induced fetal metabolic disturbances, including anaerobic metabolism, hypoxia, and acidosis, have been implicated in SB.^37^ Additionally, placental insufficiency and congenital anomalies, often associated with diabetes, can increase the risk of SB and neonatal mortality.^38^

 These complex interactions between PGDM, obesity and adverse pregnancy outcomes highlight the challenges of establishing a direct causal link through observational studies.

 While our MR analysis did not establish a direct causal relationship between PGDM and adverse pregnancy outcomes, the complex interplay of multiple factors likely contributes to this association. This relationship may be complex and influenced by non-linear, time-varying, or epigenetic factors, which are not fully captured by our linear MR analysis.^39^

 Several limitations inherent to our study warrant consideration. Firstly, the use of GWAS summary data exclusively from European populations restricts the generalizability of our findings to other ethnicities. Secondly, the MR methodology employed may not fully capture the complex and dynamic interplay between genetic predisposition, environmental factors, and lifestyle, potentially influencing the observed relationship. Moreover, certain genetic instruments exhibit low statistical power, potentially leading to nonsignificant outcomes.

## Conclusion

 We believe that there is no strong evidence to support a direct causal link between PGDM and the risks of MIS, SB, or PTB. These findings highlight the complex nature of this relationship and suggest that other factors, such as obesity, glycemic control, and underlying pathophysiological mechanisms, may mediate the increased risk observed in diabetic pregnancies. Further research is imperative to elucidate these complex interactions and to develop targeted interventions aimed at reducing the burden of adverse pregnancy outcomes in women with diabetes.

## Supplementary Files

Supplementary file 1. Comprehensive Results of IVW Method Analysis and Sensitivity Tests for SB, MIS, and PTB.

Supplementary file 2. SNPs Excluded for SB Using Cook’s Distance Analysis.

Supplementary file 3. SNPs Excluded for MIS Using Cook’s Distance Analysis.

Supplementary file 4. Details of Outlier Detection Methods and Results.
