# Supplementary material for: Pregestational Diabetes and Adverse Pregnancy Results: A Mendelian Randomization Study
Source: Arch Iran Med. 2025 Feb 1;28(2):81–7. doi: 10.34172/aim.33461 (PMC11892093; doi:10.34172/aim.33461)
Supplement: Supplementary file 1 — Comprehensive Results of IVW Method Analysis and Sensitivity Tests for SB, MIS, and PTB. [file aim-28-81-s001.pdf]

Supplementary file 1

Sedigheh Hantoushzadeh, Majid Zaki-Dizaji, Danial Habibi, Amir Hesam Saeidian, Hanifeh Mirtavoos-Mahyari, Leyla Sahebi, Mohadese Dashtkoohi, Mostafa Saeedinia, Zohreh Heidary

2024-08-10

- **Abbreviations:** (Follow tabs by these terms)
  - **DM :** Daibetes Mellitus
  - **SB :** Stillbirth
  - **MIS :** Miscarriage
  - **PR :** Preterm

[DM and SB] [DM and MIS] [DM and PR]

Introduction

- **Title:** Investigating the causality between **Maternal Diabetes Mellitus and Adverse Pregnancy Outcomes: A Mendelian Randomization Study**
  - Exposure: Diabetes Mellitus, Data downloaded from GWAS catalog (https://www.ebi.ac.uk/gwas/) | ID: GCST90132184 (https://www.ebi.ac.uk/gwas/studies/GCST90132184)
  - **Sample size**: 1,339,889 , **Number of cases**: 180,834 , **Number of controls**: 1,159,055
  - Ancestry: **European**
  - Outcome: Stillbirth, Data downloaded from IEU OpenGWAS project Database (https://gwas.mrcieu.ac.uk/) | GWAS ID: ukb-a-321 (https://gwas.mrcieu.ac.uk/datasets/ukb-a-321/)
  - **Sample size**: 178,474 , **Number of cases**: 56,172 , **Number of controls**: 122,302
  - Ancestry: **European**
  - Outcome: Miscarriage, Data downloaded from FinnGen consortium (https://storage.googleapis.com) | GWAS ID: R9-015_ABORT_SPONTAN (https://storage.googleapis.com/finngen-public-data-r9/summary_stats/finngen_R9_O15_ABORT_SPONTAN.gz)
  - **Sample size**: 166,528 , **Number of cases**: 16,906 , **Number of controls**: 149,622
  - Ancestry: **European**
  - Outcome: PR, Data downloaded from IEU OpenGWAS project Database (https://gwas.mrcieu.ac.uk/) | GWAS ID: finn-b-O15_PRETERM (https://gwas.mrcieu.ac.uk/datasets/ukb-b-15829/)
  - **Sample size**: 104,106, **Number of cases**: 5,480 , **Number of controls**: 98,626
  - Ancestry: **European**

Data Preparation

1- **Number of total SNPs in exposure:** 10,454,801 SNPs

2- **Number of Selected SNPs exposure:** 187 SNPs

3- **Number of total SNPs in outcome**: 8,818,910 SNPs

4- **Number of common variants between exposure and outcome**: 186 SNPs

5- **Number of SNPs after harmonization (action=2)** = 186 SNPs

6- **Number of SNPs after removing HLA region with exploring in HLA Genes, Nomenclature (https://hla.alleles.org/genes/index.html)** = 186 SNPs

7- **Number of SNPs after removing those that have MAF < 0.01** = 186 SNPs

8- Checking pleiotropy by PhenoScanner (http://www.phenoscanner.medschl.cam.ac.uk/):

How many SNPs have been eliminated after checking the PhenoScanner website: 5 SNPs

Checking weakness of the instruments

```
##    Min. 1st Qu.  Median    Mean 3rd Qu.    Max.
##   29.75   36.00   50.16   76.42   75.72 1415.77
```

How many SNPs have been eliminated with checking the weakness: 0 SNP

RUN an initial MR:

Initial MR analysis

id.exposure	id.outcome	outcome	exposure	method	nsnp	b	se	pval
hcWyMX	G15Z4G	outcome	exposure	MR Egger	181	-0.0044112	0.0039931	0.2707761
hcWyMX	G15Z4G	outcome	exposure	Weighted median	181	-0.0038237	0.0029768	0.1989655
hcWyMX	G15Z4G	outcome	exposure	Inverse variance weighted	181	0.0006634	0.0017601	0.7062223
hcWyMX	G15Z4G	outcome	exposure	Simple mode	181	-0.0041269	0.0069895	0.5556395
hcWyMX	G15Z4G	outcome	exposure	Weighted mode	181	-0.0054408	0.0041052	0.1867307

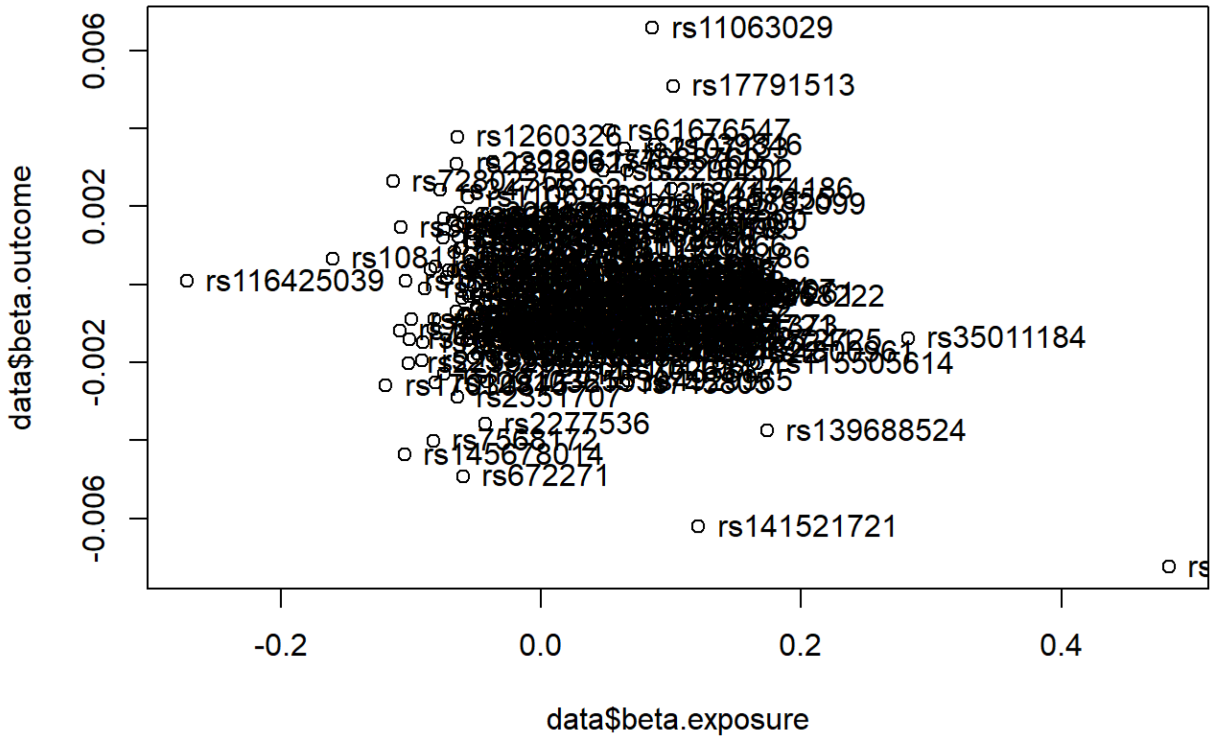

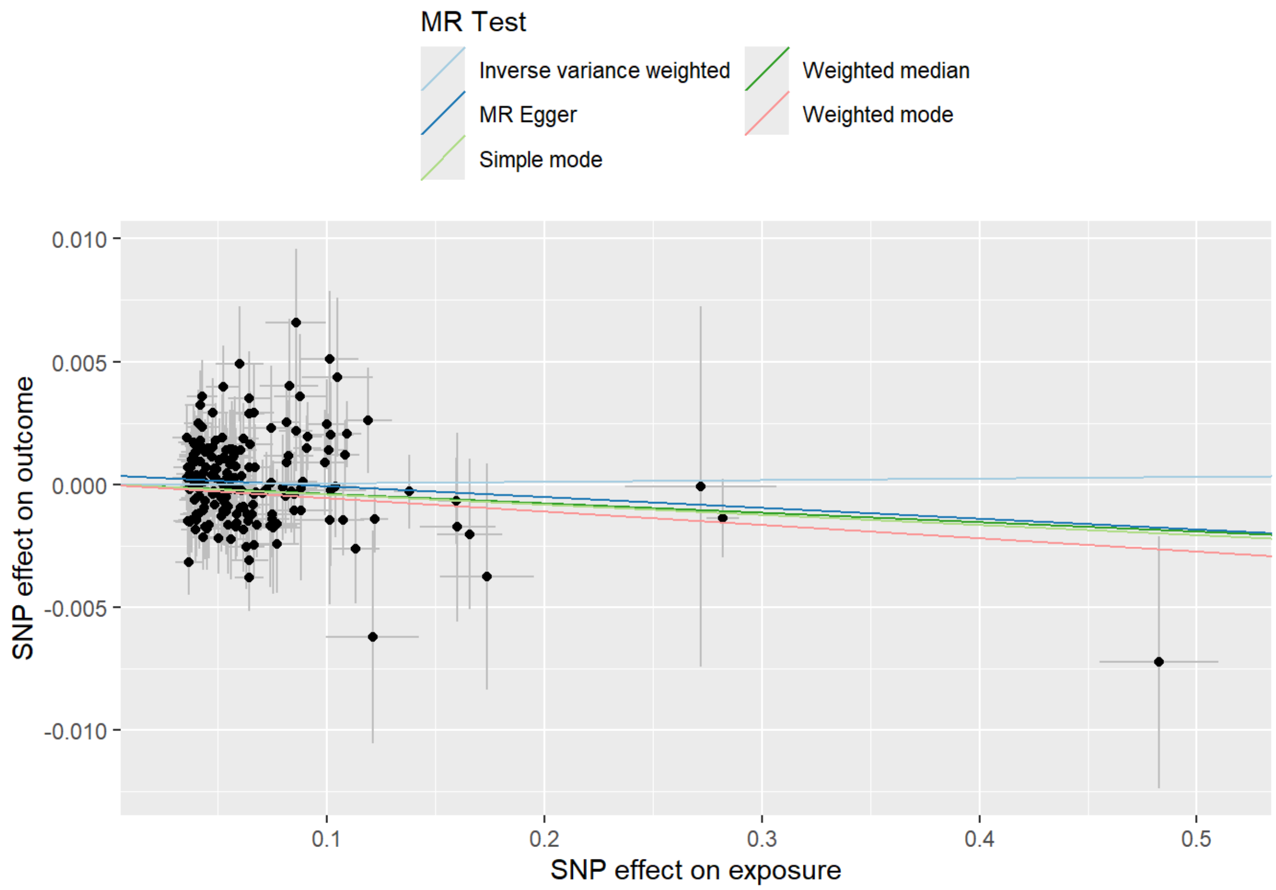

Heterogeneity testing

id.exposure	id.outcome	outcome	exposure	method	Q	Q_df	Q_pval
hcWyMX	G15Z4G	outcome	exposure	MR Egger	170.3396	179	0.6663752

id.exposure	id.outcome	outcome	exposure	method	Q	Q_df	Q_pval
hcWyMX	G15Z4G	outcome	exposure	Inverse variance weighted	172.3441	180	0.6458346

pleiotropy testing

id.exposure	id.outcome	outcome	exposure	egger_intercept	se	pval
hcWyMX	G15Z4G	outcome	exposure	0.0003747	0.0002646	0.1585748

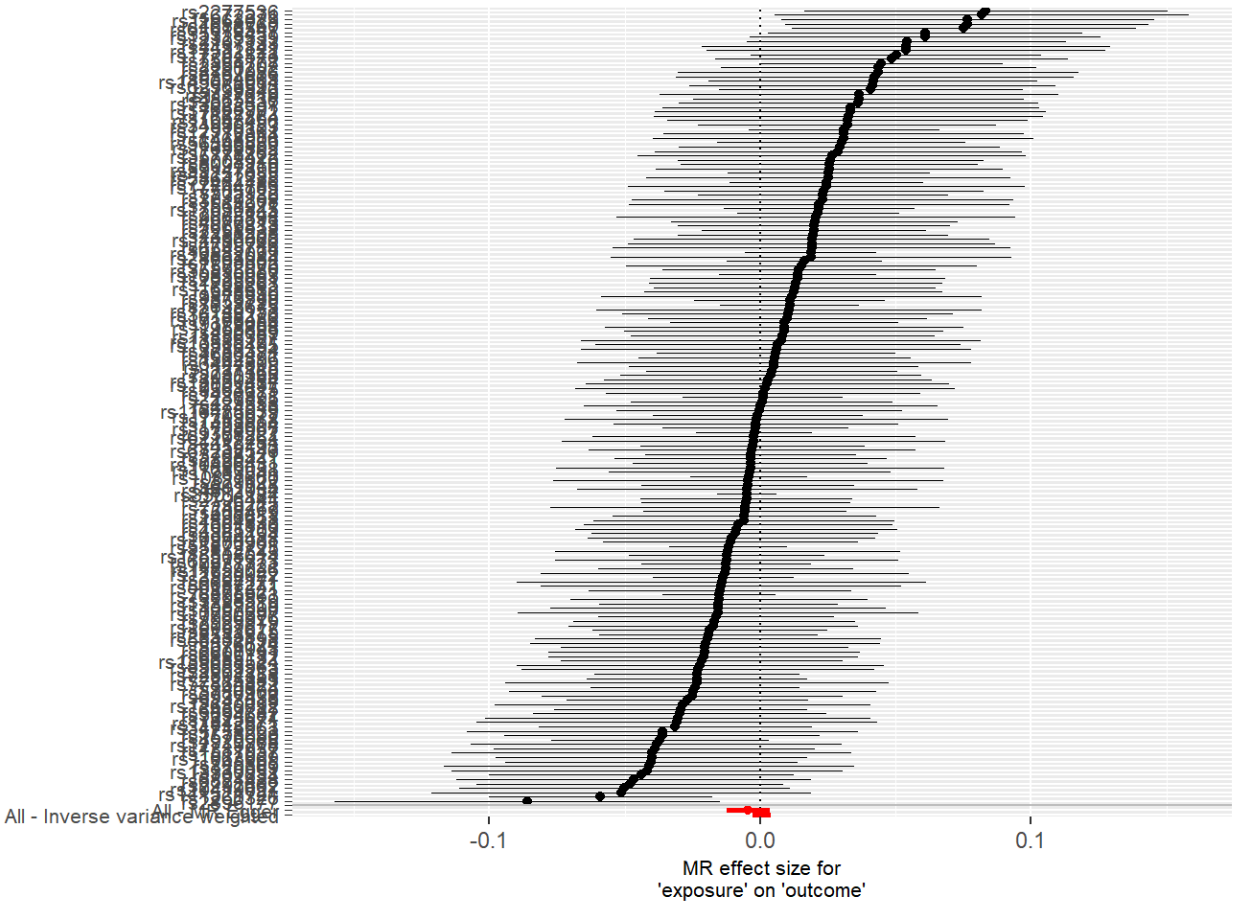
All - Inverse variance weighted
-0.1
0.0
0.1
MR effect size for
'exposure' on 'outcome'

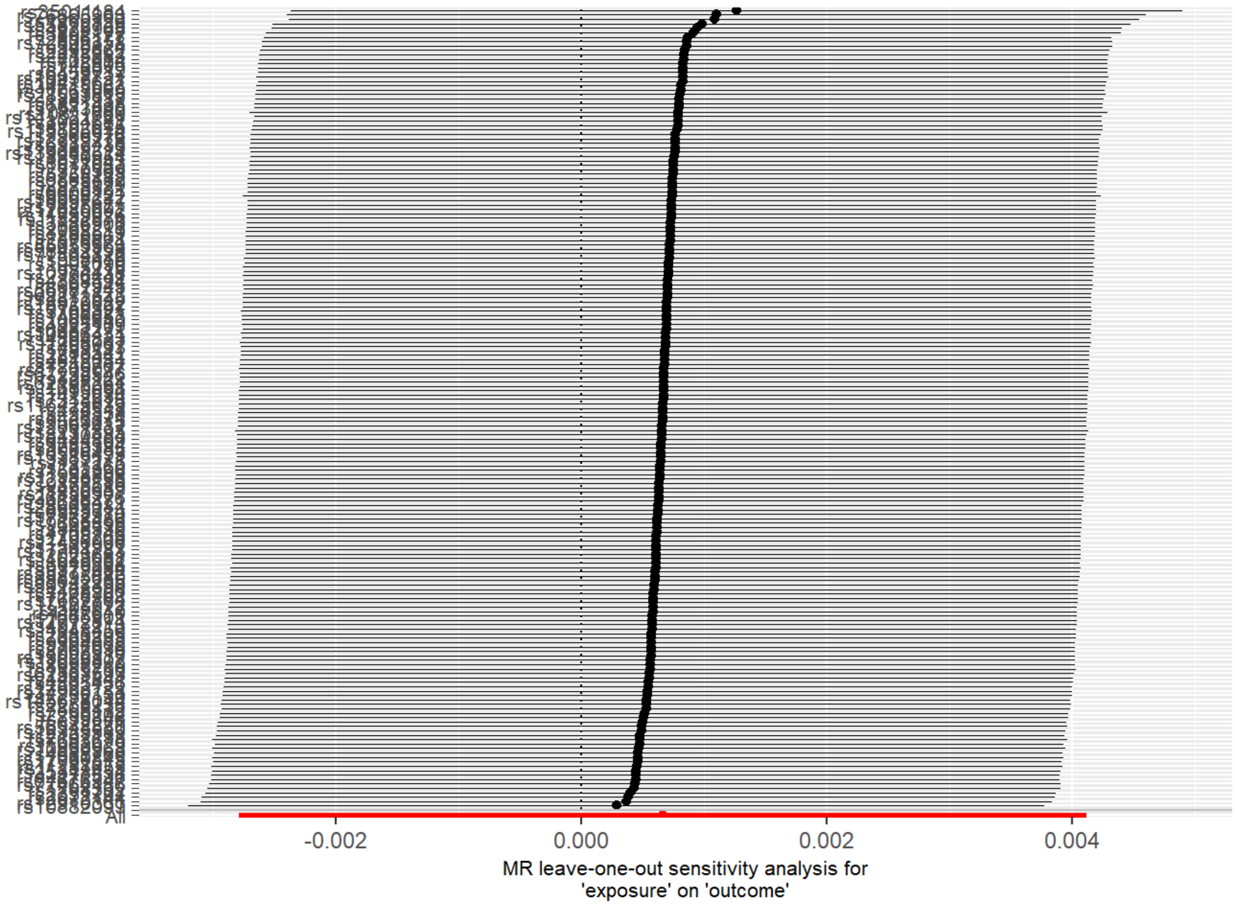
All
-0.002
0.000
0.002
0.004
MR leave-one-out sensitivity analysis for
'exposure' on 'outcome'

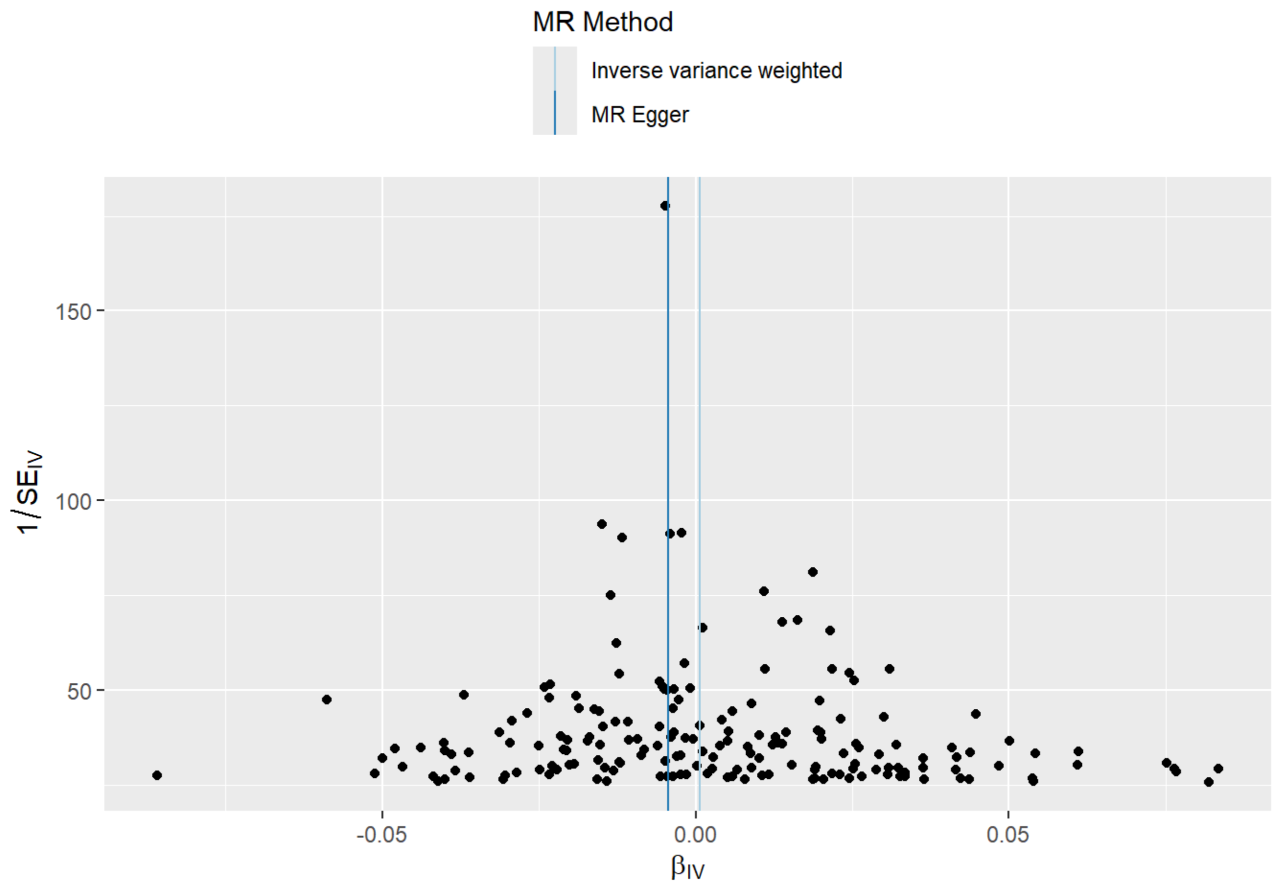

Testing Outlier with PRESSO test

```
## $`Main MR results`
##        Exposure       MR Analysis Causal Estimate          Sd    T-stat
## 1 beta.exposure                Raw    0.0006634327 0.001722237 0.3852157
## 2 beta.exposure Outlier-corrected              NA          NA        NA
##     P-value
## 1 0.7005324
## 2        NA
##
## $`MR-PRESSO results`
## $`MR-PRESSO results`$`Global Test`
## $`MR-PRESSO results`$`Global Test`$RSSobs
## [1] 174.343
##
## $`MR-PRESSO results`$`Global Test`$Pvalue
## [1] 0.649
```

MR analysis after excluding SNPs detected by MRPRESSO

id.exposure	id.outcome	outcome	exposure	method	nsnp	b	se	pval
hcWyMX	G15Z4G	outcome	exposure	MR Egger	181	-0.0044112	0.0039931	0.2707761
hcWyMX	G15Z4G	outcome	exposure	Weighted median	181	-0.0038237	0.0030556	0.2108097

id.exposure	id.outcome	outcome	exposure	method	nsnp	b	se	pval
hcWyMX	G15Z4G	outcome	exposure	Inverse variance weighted	181	0.0006634	0.0017601	0.7062223
hcWyMX	G15Z4G	outcome	exposure	Simple mode	181	-0.0041269	0.0076605	0.5907481
hcWyMX	G15Z4G	outcome	exposure	Weighted mode	181	-0.0054408	0.0040959	0.1857391

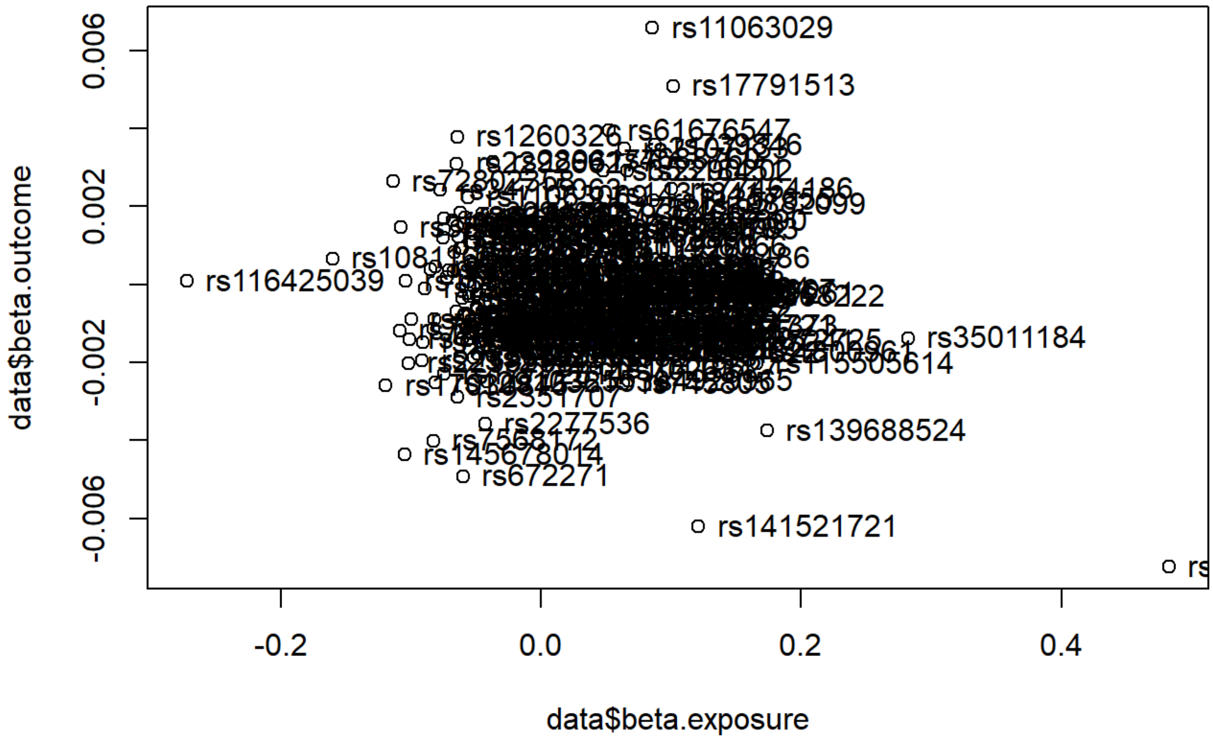

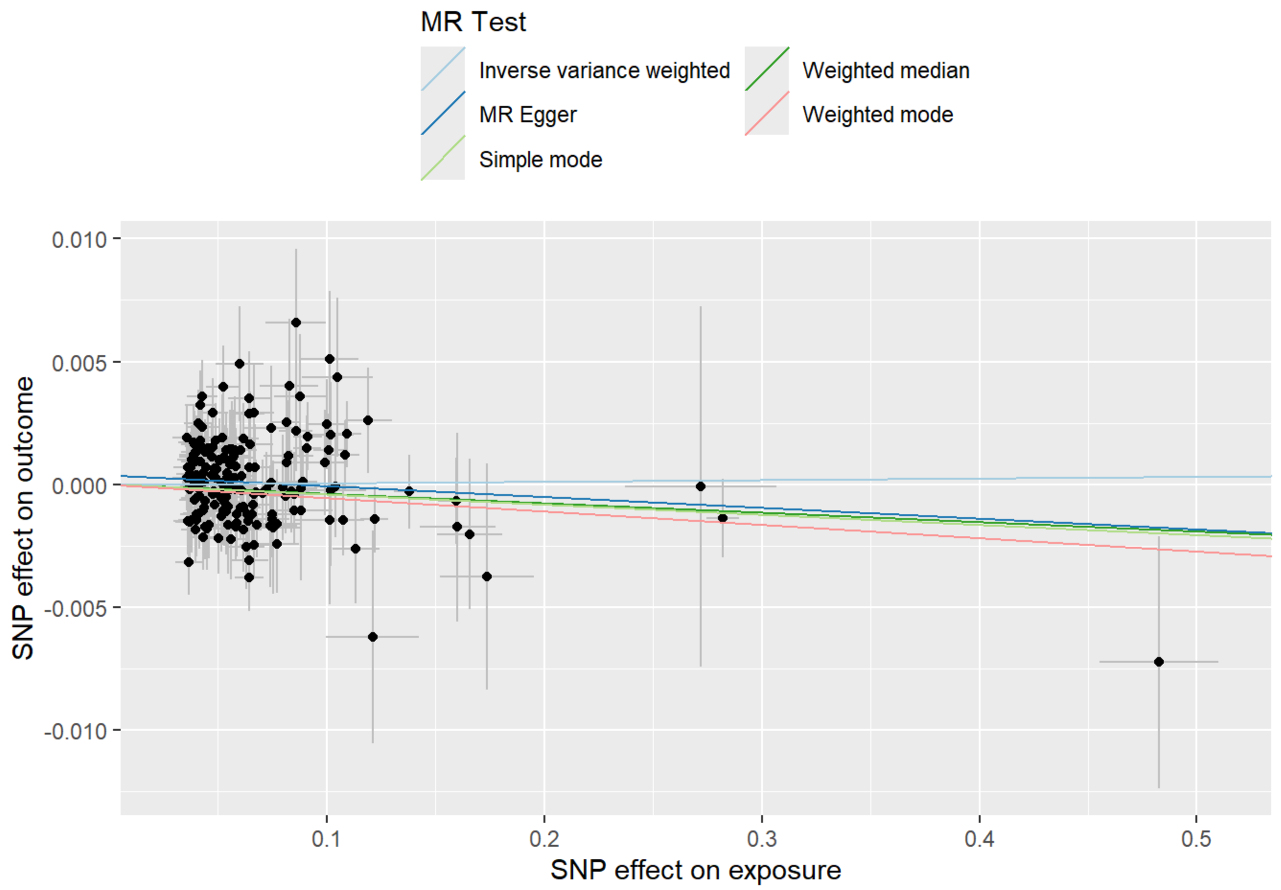

Heterogeneity testing

id.exposure	id.outcome	outcome	exposure	method	Q	Q_df	Q_pval
hcWyMX	G15Z4G	outcome	exposure	MR Egger	170.3396	179	0.6663752

id.exposure	id.outcome	outcome	exposure	method	Q	Q_df	Q_pval
hcWyMX	G15Z4G	outcome	exposure	Inverse variance weighted	172.3441	180	0.6458346

pleiotropy testing

id.exposure	id.outcome	outcome	exposure	egger_intercept	se	pval
hcWyMX	G15Z4G	outcome	exposure	0.0003747	0.0002646	0.1585748

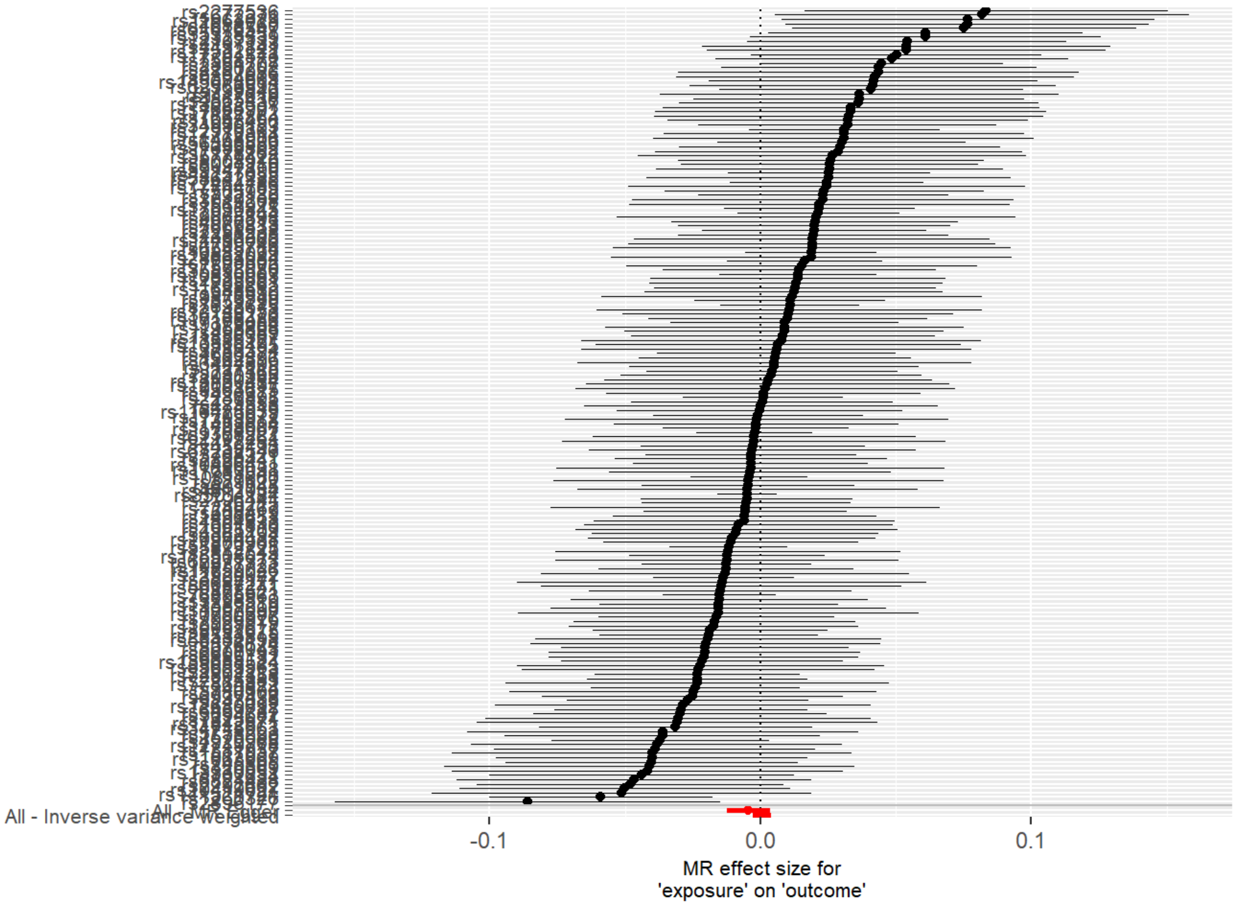
All - Inverse variance weighted
-0.1
0.0
0.1
MR effect size for
'exposure' on 'outcome'

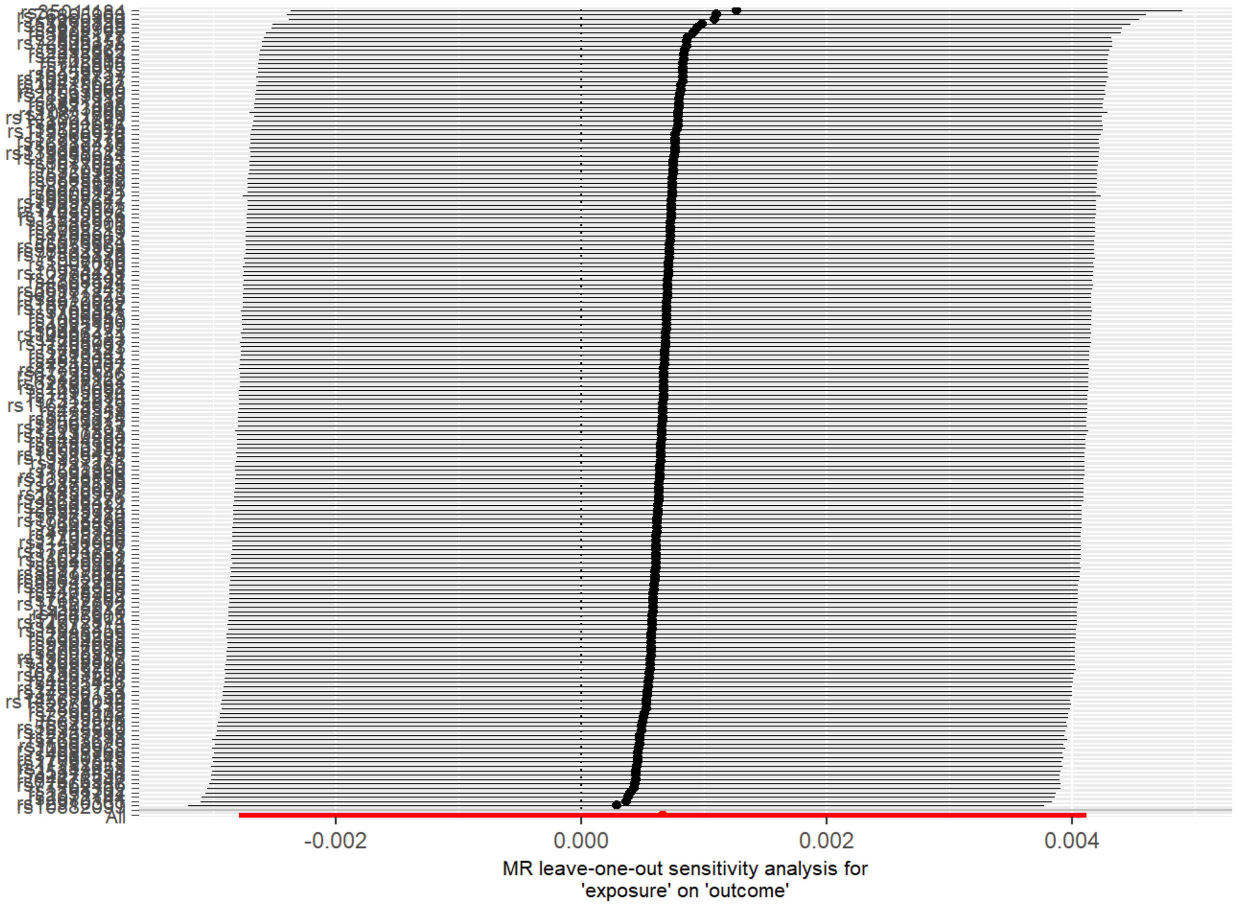
All
-0.002
0.000
0.002
0.004
MR leave-one-out sensitivity analysis for
'exposure' on 'outcome'

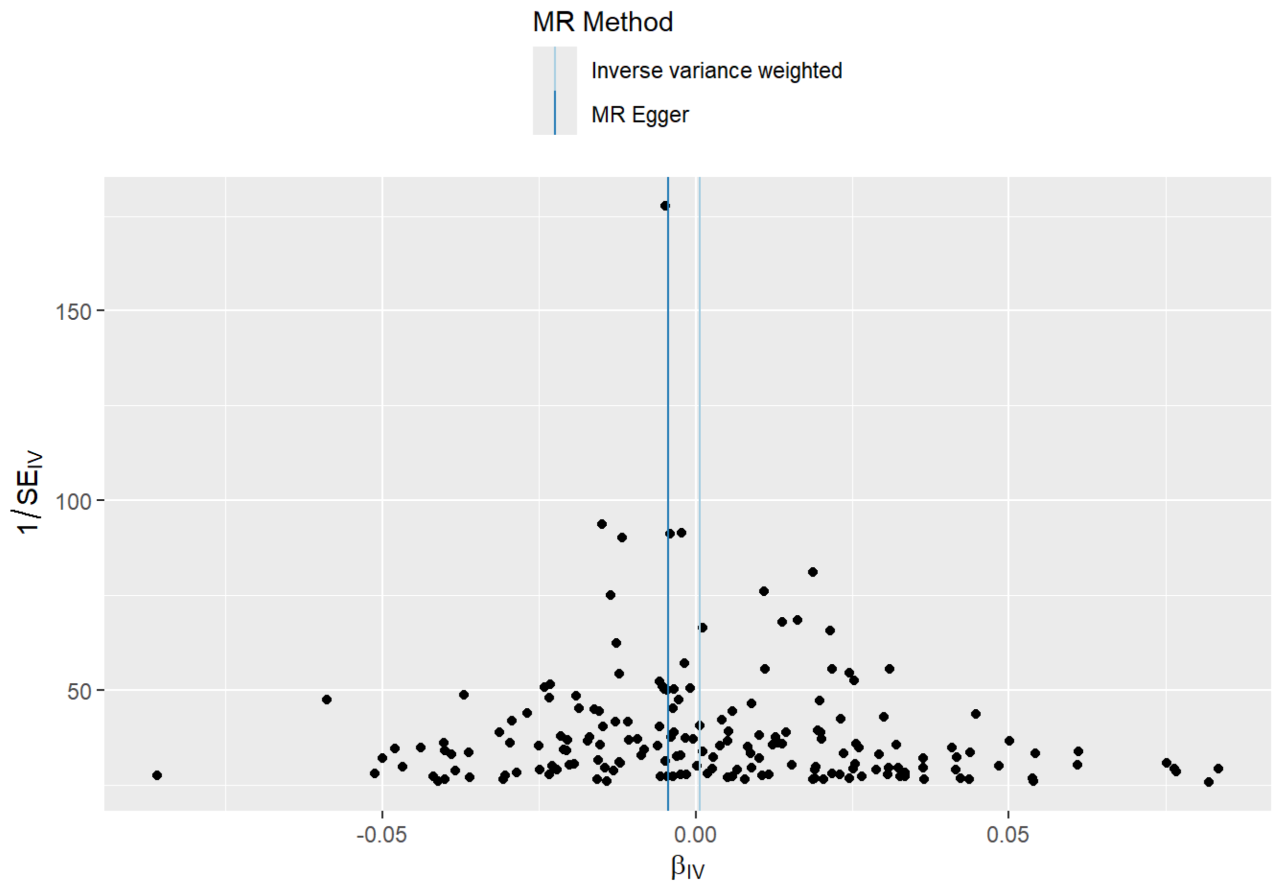

Radial test

```
##
## Radial IVW
##
##                        Estimate   Std.Error   t value  Pr(>|t|)
## Effect (Mod.2nd) 0.0006634341 0.001722237 0.3852166 0.7000770
## Iterative        0.0006634341 0.001722237 0.3852166 0.7000770
## Exact (FE)       0.0006746442 0.001760083 0.3833024 0.7014956
## Exact (RE)       0.0006721110 0.001784785 0.3765781 0.7069307
##
##
## Residual standard error: 0.978 on 180 degrees of freedom
##
## F-statistic: 0.15 on 1 and 180 DF, p-value: 0.701
## Q-Statistic for heterogeneity: 172.3423 on 180 DF , p-value: 0.6458702
##
##  No significant outliers
## Number of iterations = 2
```

```
## [1] "No significant outliers"
```

MR analysis after excluding SNPs detected by MRPRESSO

id.exposure	id.outcome	outcome	exposure	method	nsnp	b	se	pval
hcWyMX	G15Z4G	outcome	exposure	MR Egger	181	-0.0044112	0.0039931	0.2707761

id.exposure	id.outcome	outcome	exposure	method	nsnp	b	se	pval
hcWyMX	G15Z4G	outcome	exposure	Weighted median	181	-0.0038237	0.0030557	0.2108177
hcWyMX	G15Z4G	outcome	exposure	Inverse variance weighted	181	0.0006634	0.0017601	0.7062223
hcWyMX	G15Z4G	outcome	exposure	Simple mode	181	-0.0041269	0.0071317	0.5635392
hcWyMX	G15Z4G	outcome	exposure	Weighted mode	181	-0.0054408	0.0039035	0.1650822

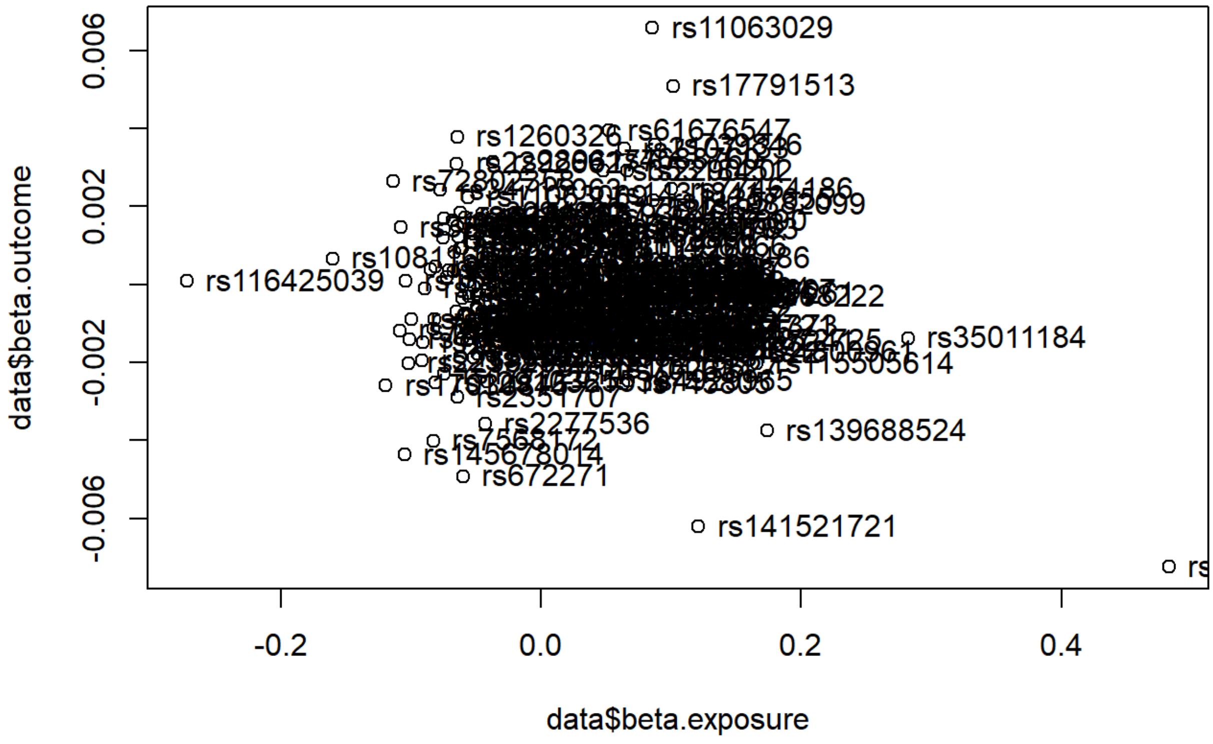

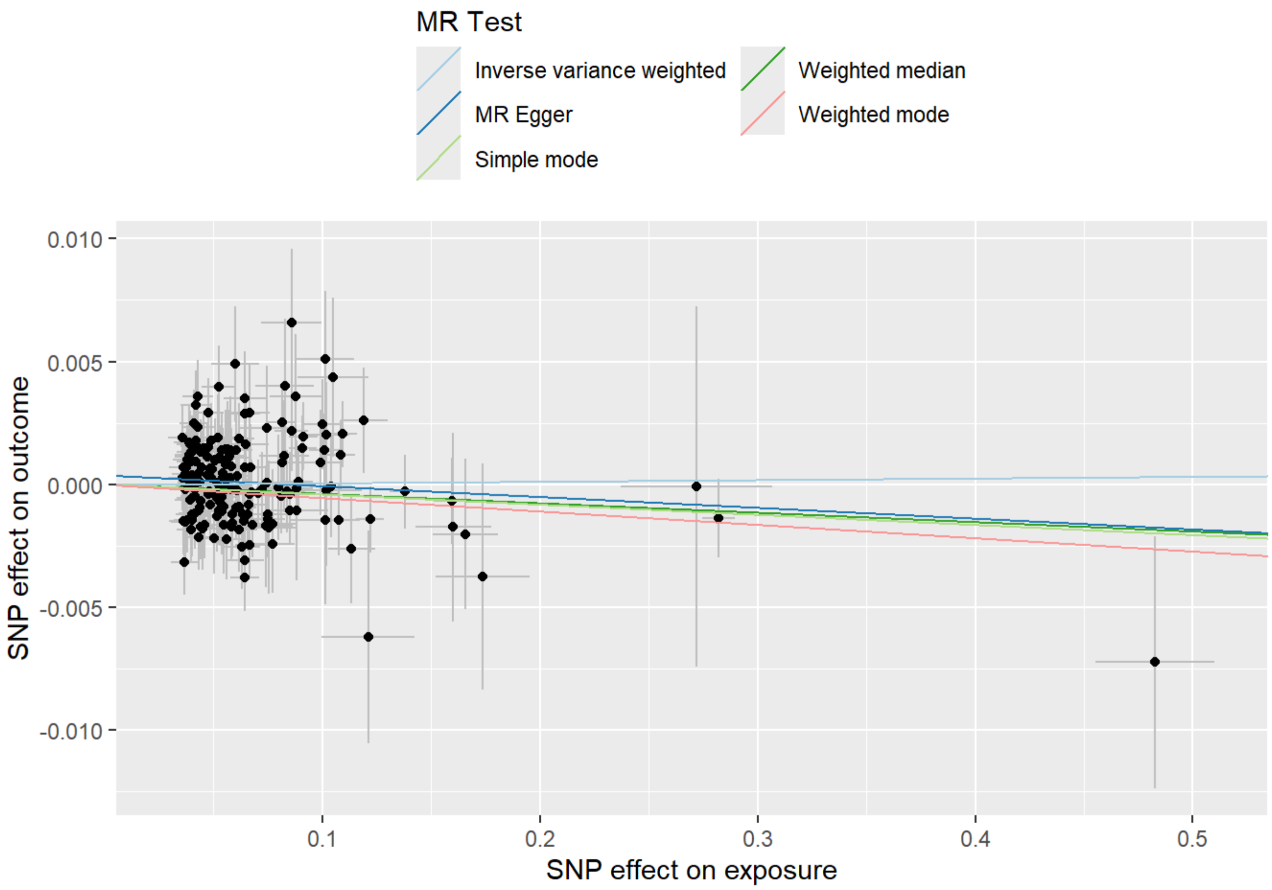

Heterogeneity testing

id.exposure	id.outcome	outcome	exposure	method	Q	Q_df	Q_pval
hcWyMX	G15Z4G	outcome	exposure	MR Egger	170.3396	179	0.6663752

id.exposure	id.outcome	outcome	exposure	method	Q	Q_df	Q_pval
hcWyMX	G15Z4G	outcome	exposure	Inverse variance weighted	172.3441	180	0.6458346

pleiotropy testing

id.exposure	id.outcome	outcome	exposure	egger_intercept	se	pval
hcWyMX	G15Z4G	outcome	exposure	0.0003747	0.0002646	0.1585748

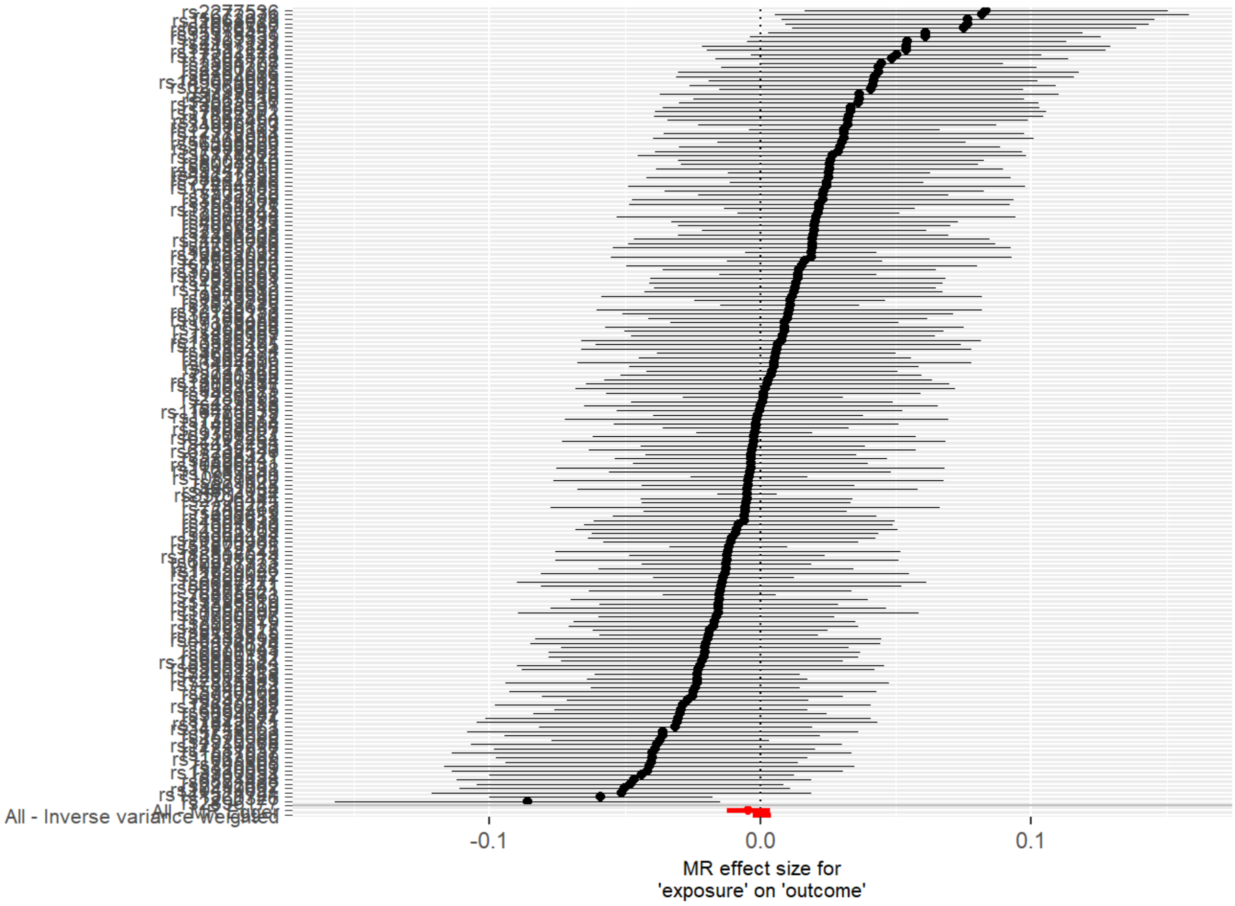
All - Inverse variance weighted
-0.1
0.0
0.1
MR effect size for
'exposure' on 'outcome'

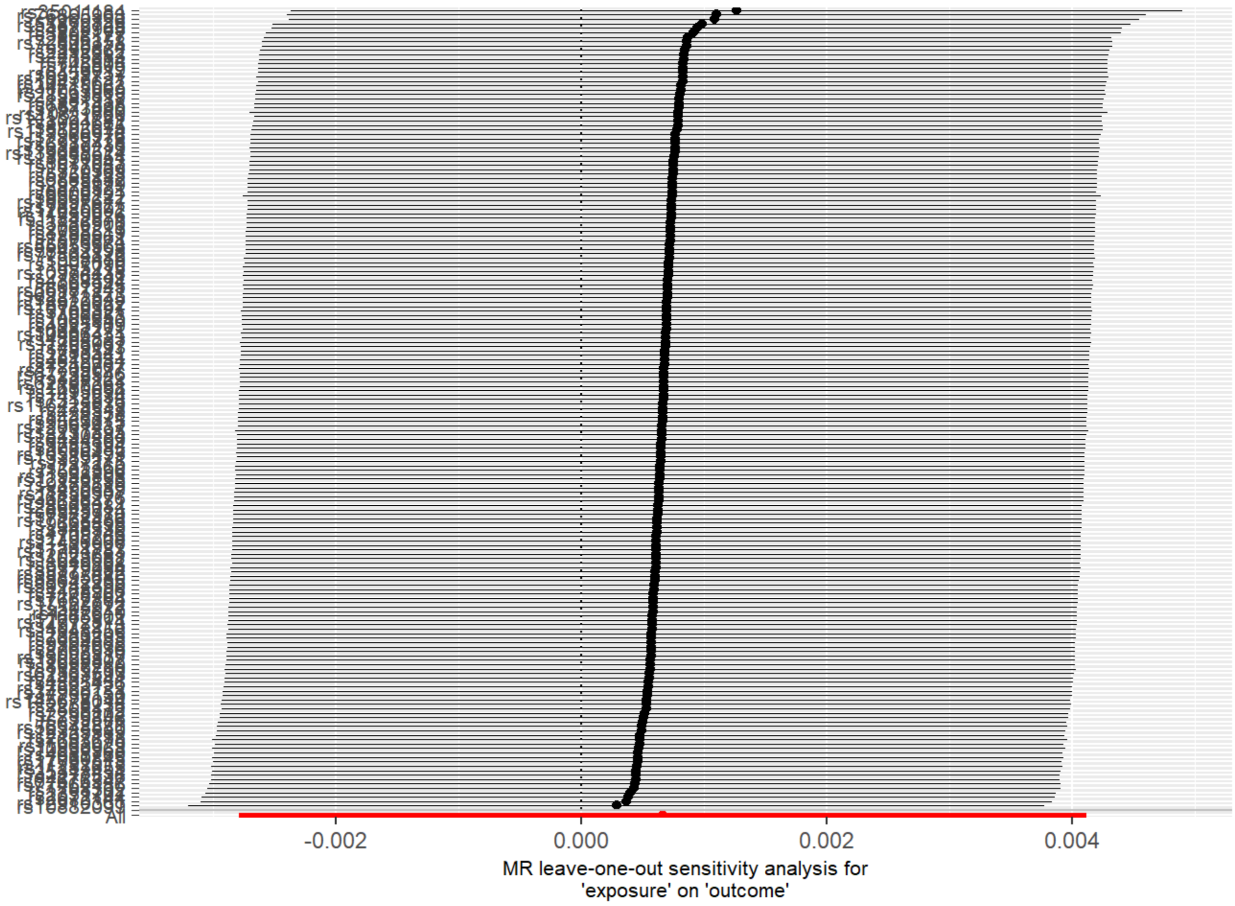
All
-0.002
0.000
0.002
0.004
MR leave-one-out sensitivity analysis for
'exposure' on 'outcome'

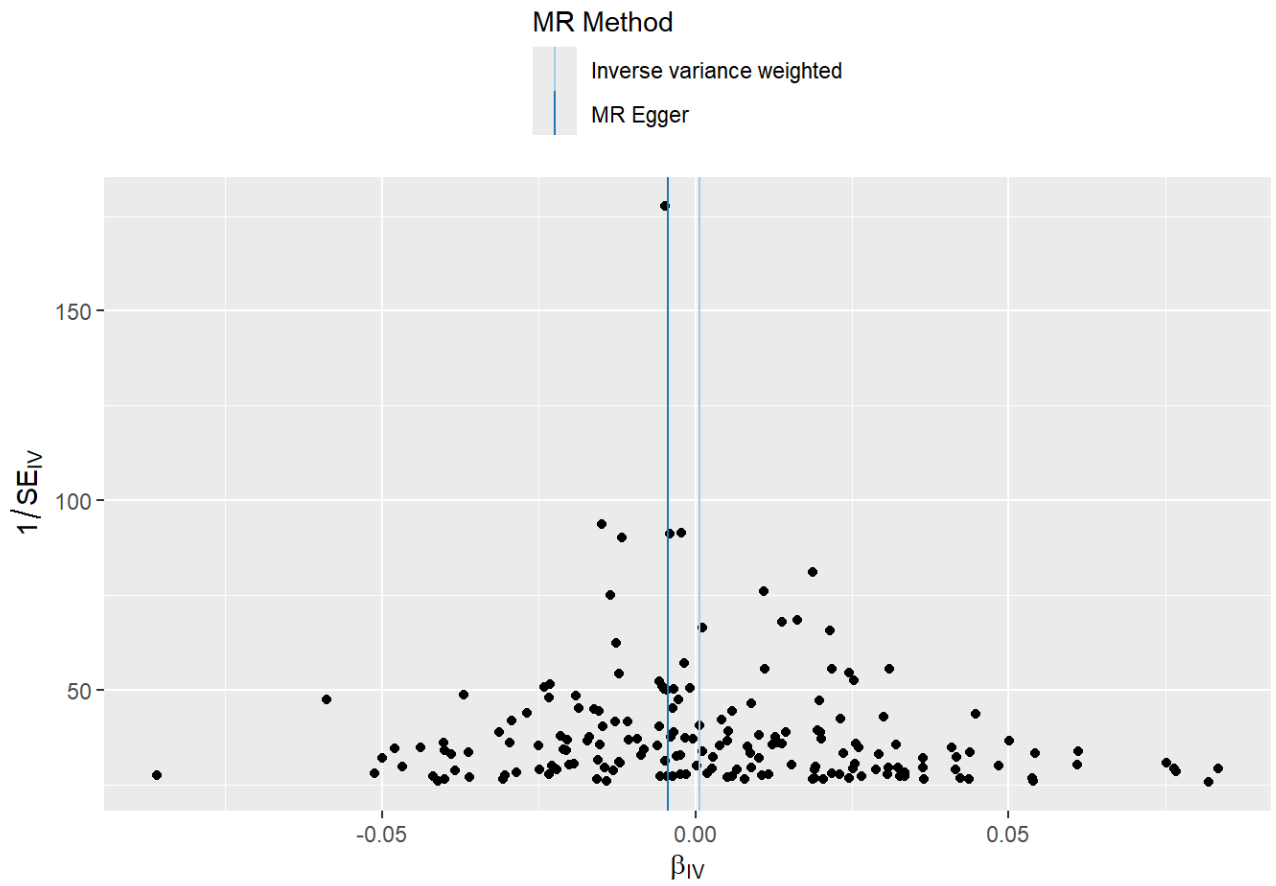

Cook's distance

In statistics, Cook's distance or Cook's D is a commonly used estimate of the influence of a data point when performing a least-squares regression analysis.[1] In a practical ordinary least squares analysis, Cook's distance can be used in several ways:

1- To indicate influential data points that are particularly worth checking for validity.

2- To indicate regions of the design space where it would be good to be able to obtain more data points.

It is named after the American statistician R. Dennis Cook, who introduced the concept in 1977.

Refernce (https://link.springer.com/article/10.1007/s10654-017-0255-x)

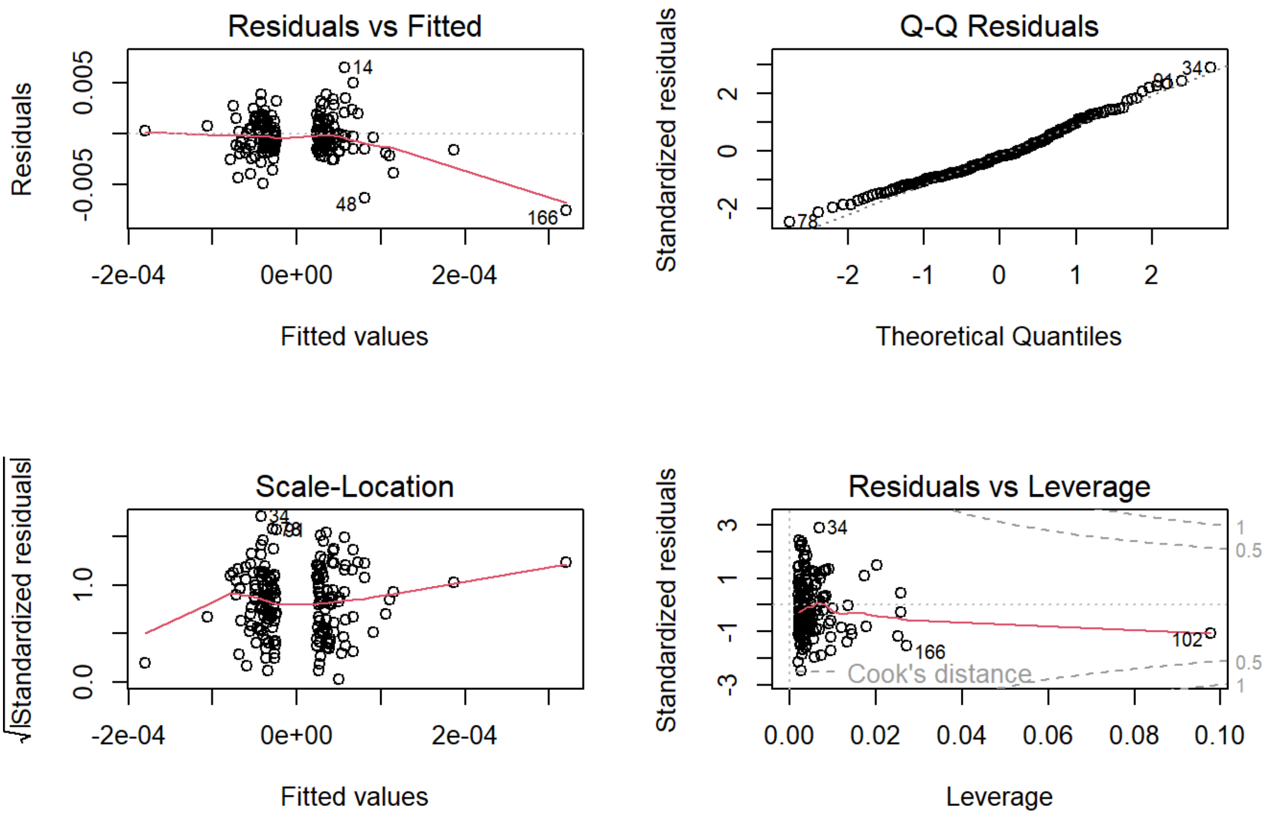

Run After deleting new outlier: Final Results:

MR analysis after deleting outliers

id.exposure	id.outcome	outcome	exposure	method	nsnp	b	se	pval
hcWyMX	G15Z4G	outcome	exposure	MR Egger	172	-0.0040603	0.0042636	0.3422936
hcWyMX	G15Z4G	outcome	exposure	Weighted median	172	-0.0035997	0.0032115	0.2623374
hcWyMX	G15Z4G	outcome	exposure	Inverse variance weighted	172	-0.0000178	0.0018069	0.9921388
hcWyMX	G15Z4G	outcome	exposure	Simple mode	172	-0.0036543	0.0070403	0.6043901
hcWyMX	G15Z4G	outcome	exposure	Weighted mode	172	-0.0049495	0.0043896	0.2610920

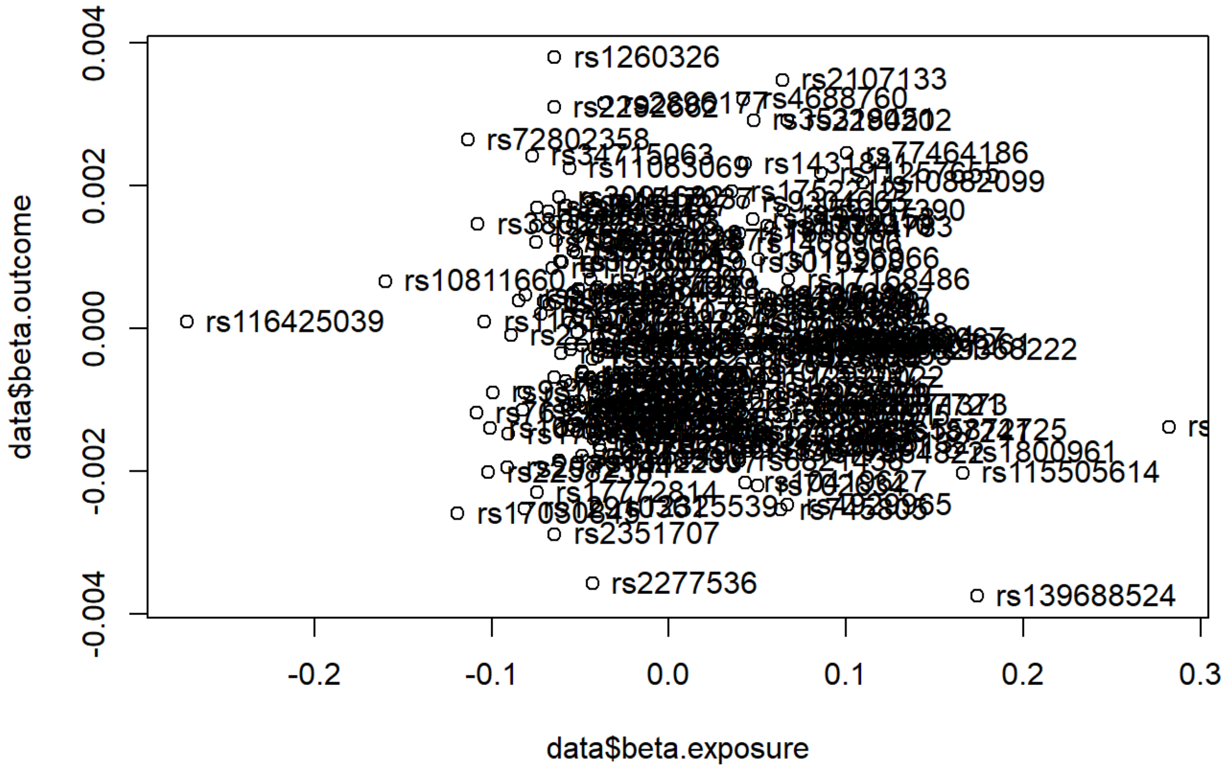

Heterogeneity testing

id.exposure	id.outcome	outcome	exposure	method	Q	Q_df	Q_pval
hcWyMX	G15Z4G	outcome	exposure	MR Egger	143.4608	170	0.9312530
hcWyMX	G15Z4G	outcome	exposure	Inverse variance weighted	144.5566	171	0.9298286

pleiotropy testing

id.exposure	id.outcome	outcome	exposure	egger_intercept	se	pval
hcWyMX	G15Z4G	outcome	exposure	0.0002906	0.0002776	0.296687

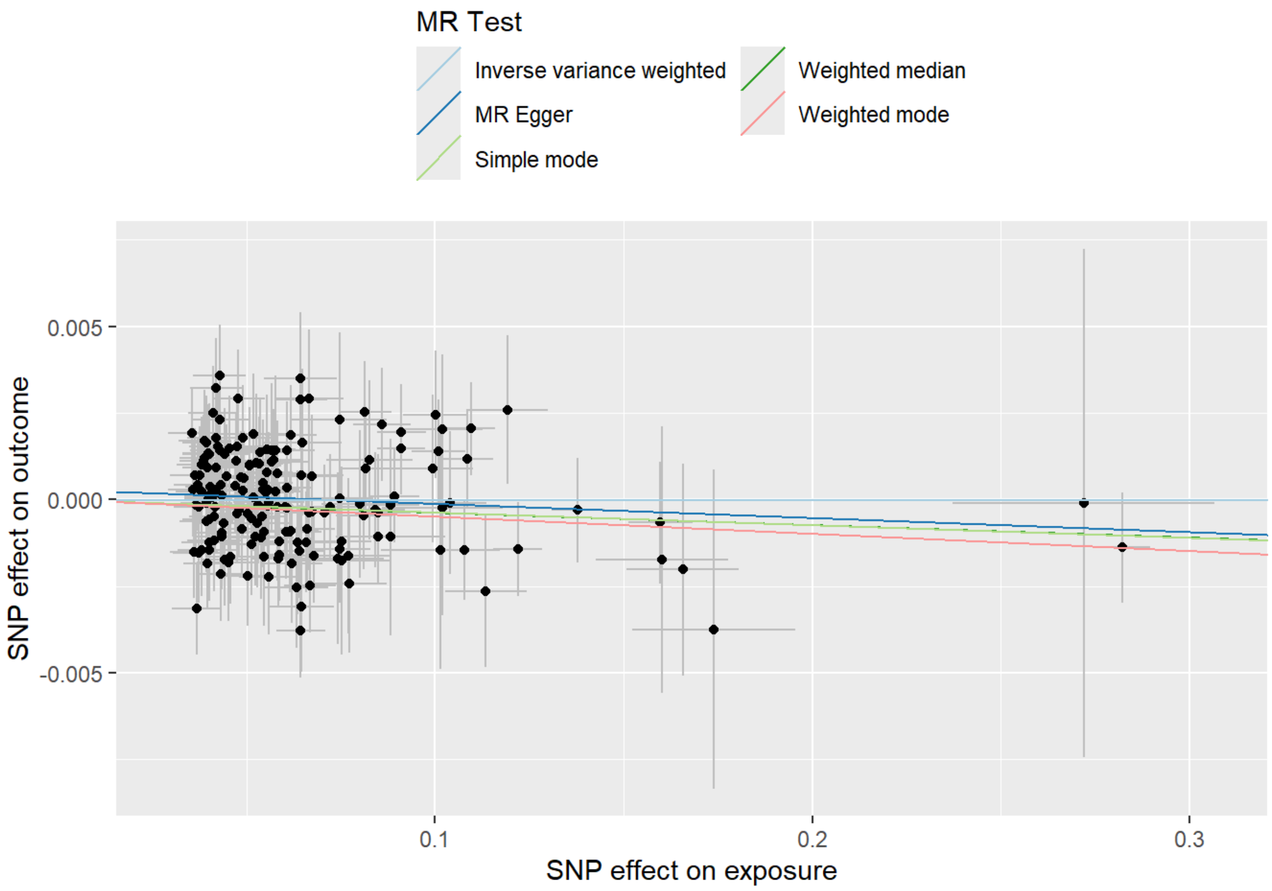
MR Test
Inverse variance weighted
Weighted median
MR Egger
Weighted mode
Simple mode
0.005
0.000
-0.005
SNP effect on outcome
0.1
0.2
0.3
SNP effect on exposure

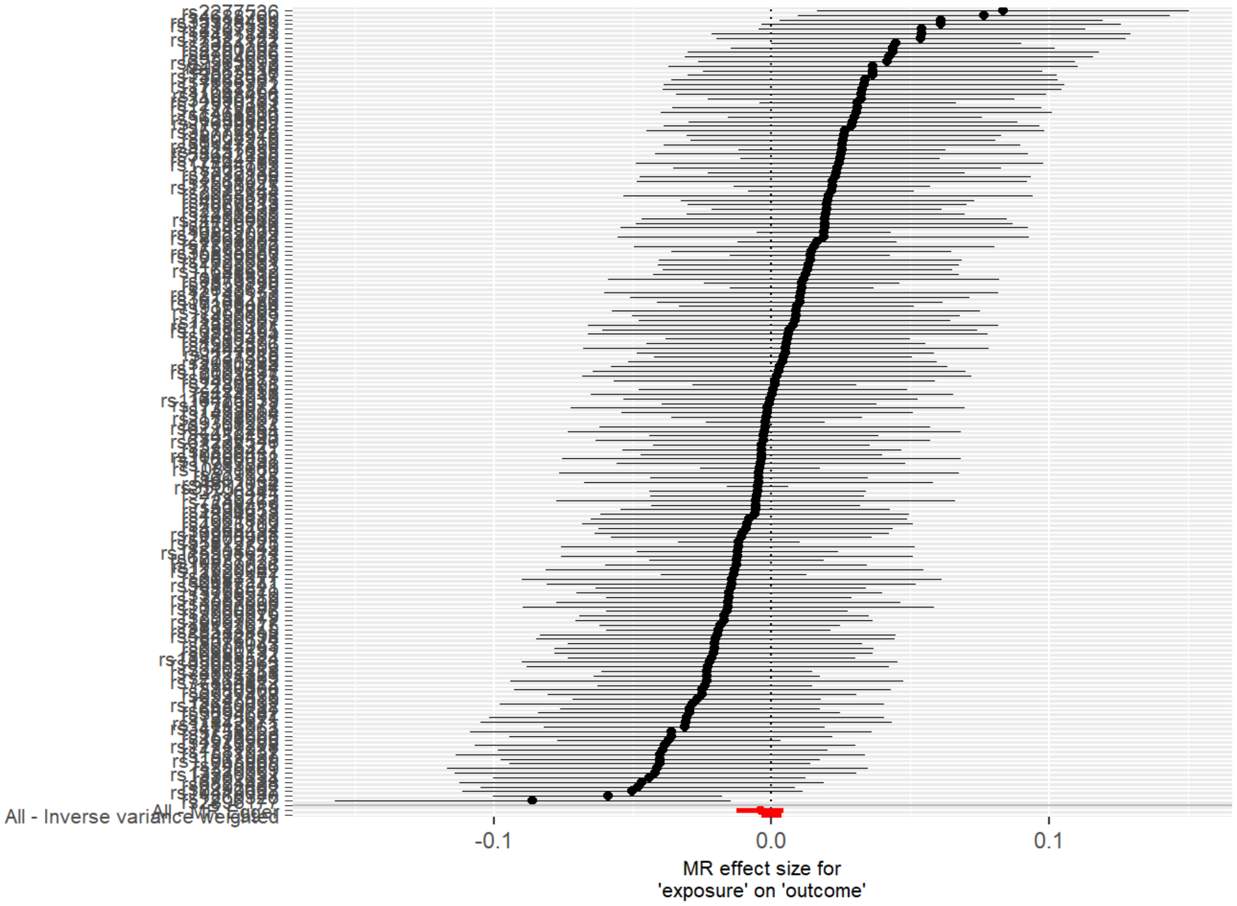
All - Inverse variance weighted
All - MR Egger
-0.1
0.0
0.1
MR effect size for
'exposure' on 'outcome'

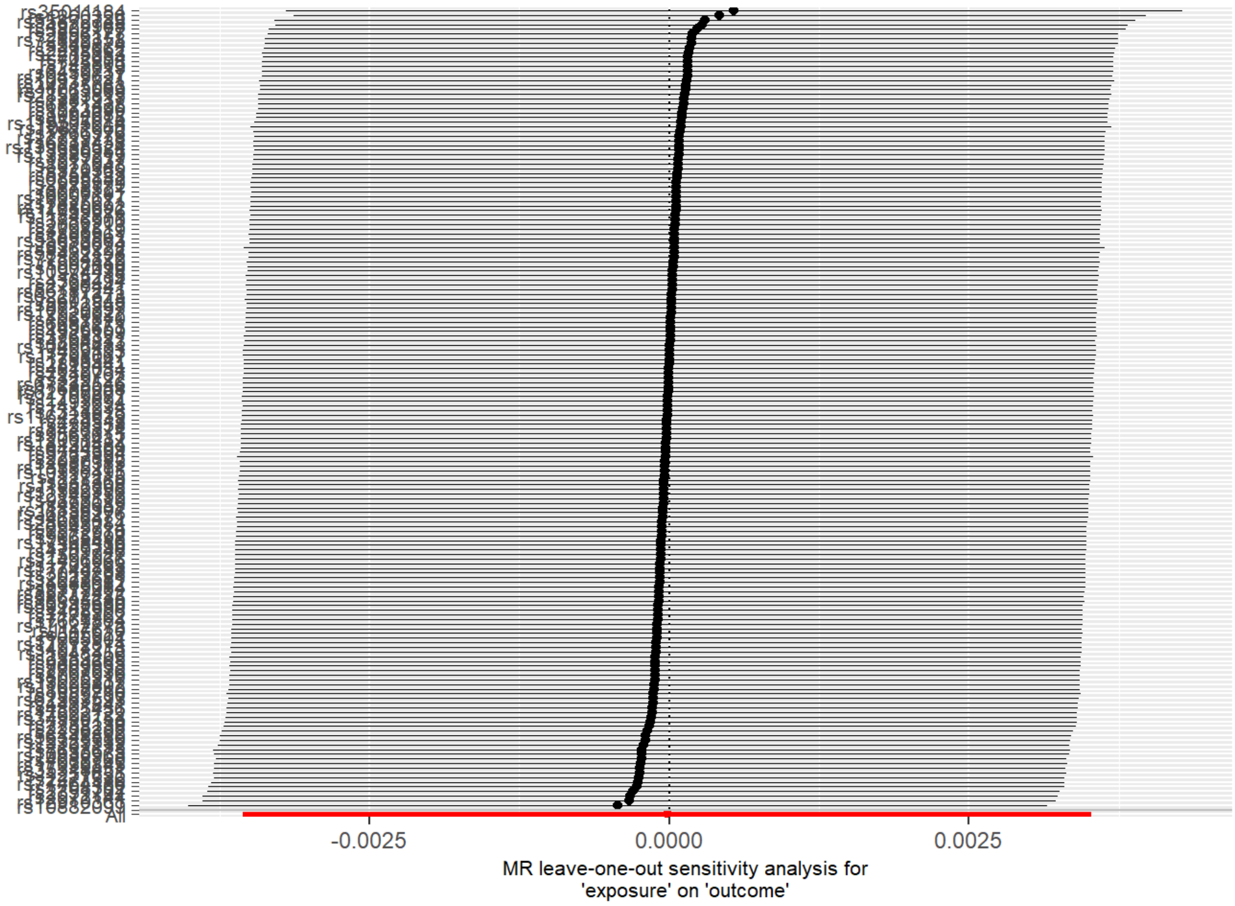
All
-0.0025
0.0000
0.0025
MR leave-one-out sensitivity analysis for
'exposure' on 'outcome'

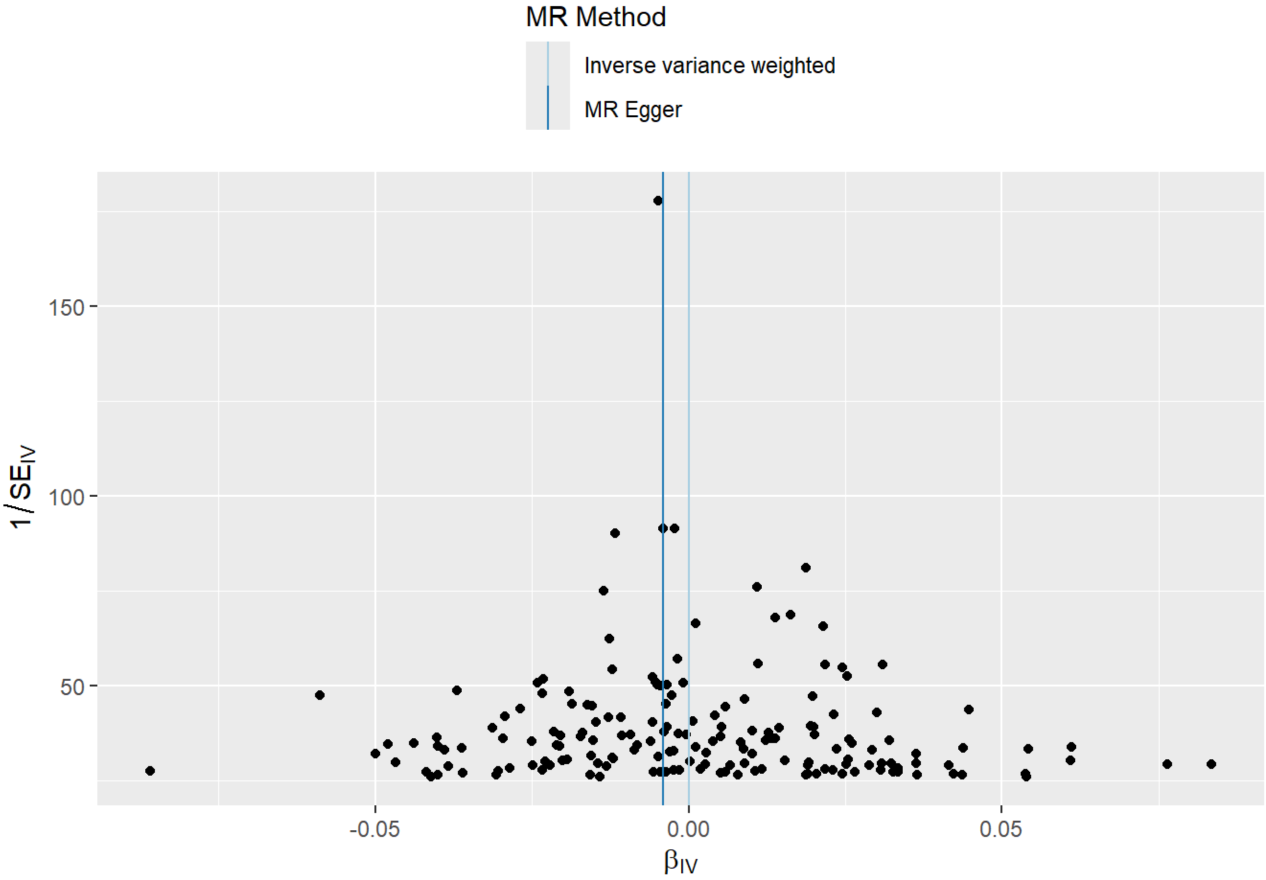
MR Method
Inverse variance weighted
MR Egger
150
100
50
-0.05
0.00
0.05
$1/SE_{IV}$
β_{IV}

Sensitivity analyses with MendelianRandomization Package

```
##
## Inverse-variance weighted method
## (variants uncorrelated, random-effect model)
##
## Number of Variants : 172
##
## ------------------------------------------------------------------
##  Method Estimate Std Error  95% CI       p-value
##     IVW    0.000     0.002 -0.004, 0.004   0.992
## ------------------------------------------------------------------
## Residual standard error =  0.919
## Residual standard error is set to 1 in calculation of confidence interval when its esti
mate is less than 1.
## Heterogeneity test statistic (Cochran's Q) = 144.5566 on 171 degrees of freedom, (p-val
ue = 0.9298). I^2 = 0.0%.
## F statistic = 76.7.
```

```
##                      Method Estimate Std Error 95% CI        P-value
##               Simple median   -0.001     0.003  -0.007 0.004   0.685
##             Weighted median   -0.004     0.003  -0.010 0.003   0.258
##  Penalized weighted median   -0.004     0.003  -0.010 0.003   0.258
##
##                         IVW    0.000     0.002  -0.004 0.004   0.992
##               Penalized IVW    0.000     0.002  -0.003 0.004   0.932
##                  Robust IVW    0.000     0.002  -0.004 0.004   0.924
##        Penalized robust IVW    0.000     0.002  -0.004 0.004   0.928
##
##                    MR-Egger   -0.004     0.004  -0.012 0.004   0.341
##                 (intercept)    0.000     0.000   0.000 0.001   0.295
##          Penalized MR-Egger   -0.004     0.004  -0.012 0.004   0.354
##                 (intercept)    0.000     0.000   0.000 0.001   0.281
##             Robust MR-Egger   -0.005     0.003  -0.011 0.001   0.133
##                 (intercept)    0.000     0.000   0.000 0.001   0.142
##  Penalized robust MR-Egger   -0.005     0.003  -0.011 0.001   0.134
##                 (intercept)    0.000     0.000   0.000 0.001   0.140
```

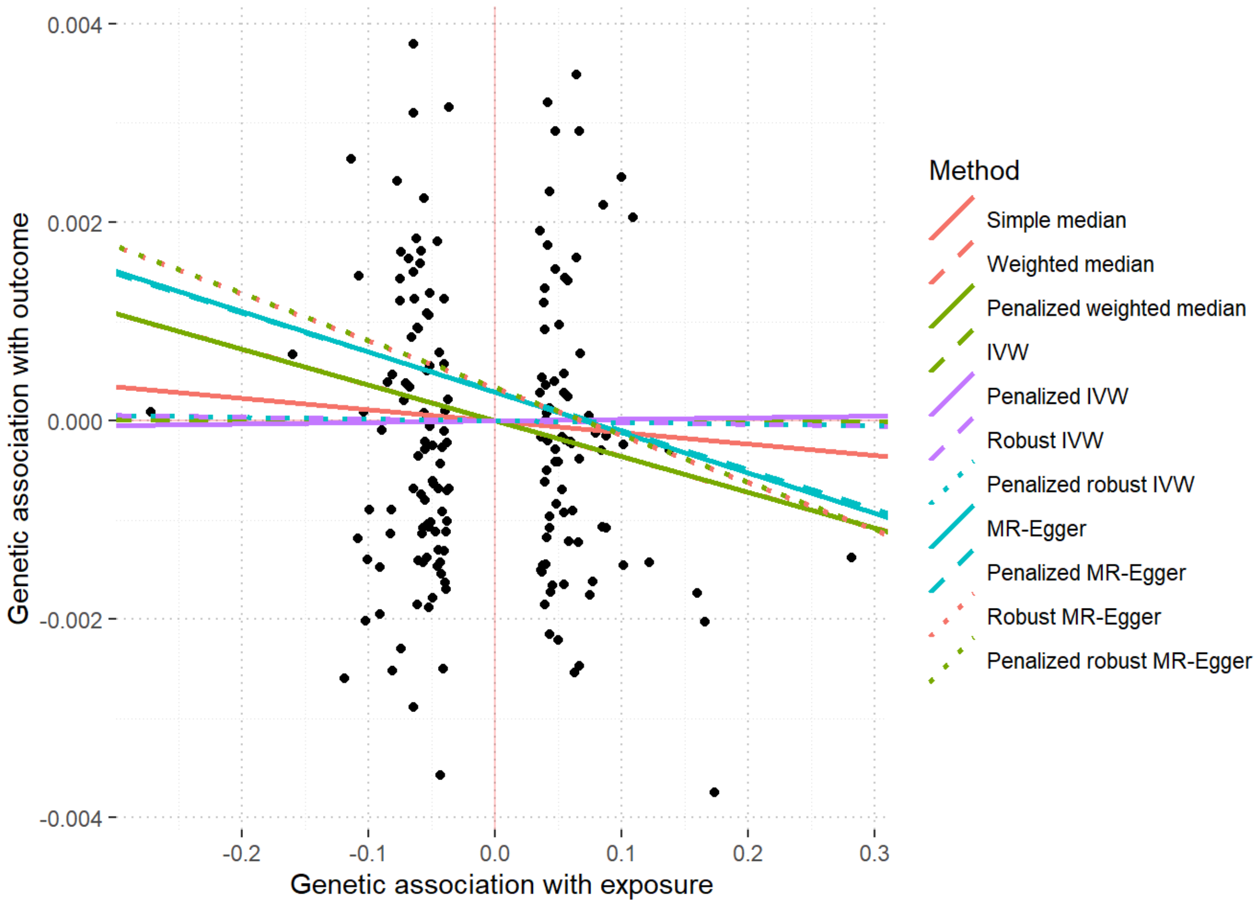
0.004
0.002
0.000
-0.002
-0.004
-0.2
-0.1
0.0
0.1
0.2
0.3
Genetic association with outcome
Genetic association with exposure
Method
Simple median
Weighted median
Penalized weighted median
IVW
Penalized IVW
Robust IVW
Penalized robust IVW
MR-Egger
Penalized MR-Egger
Robust MR-Egger
Penalized robust MR-Egger

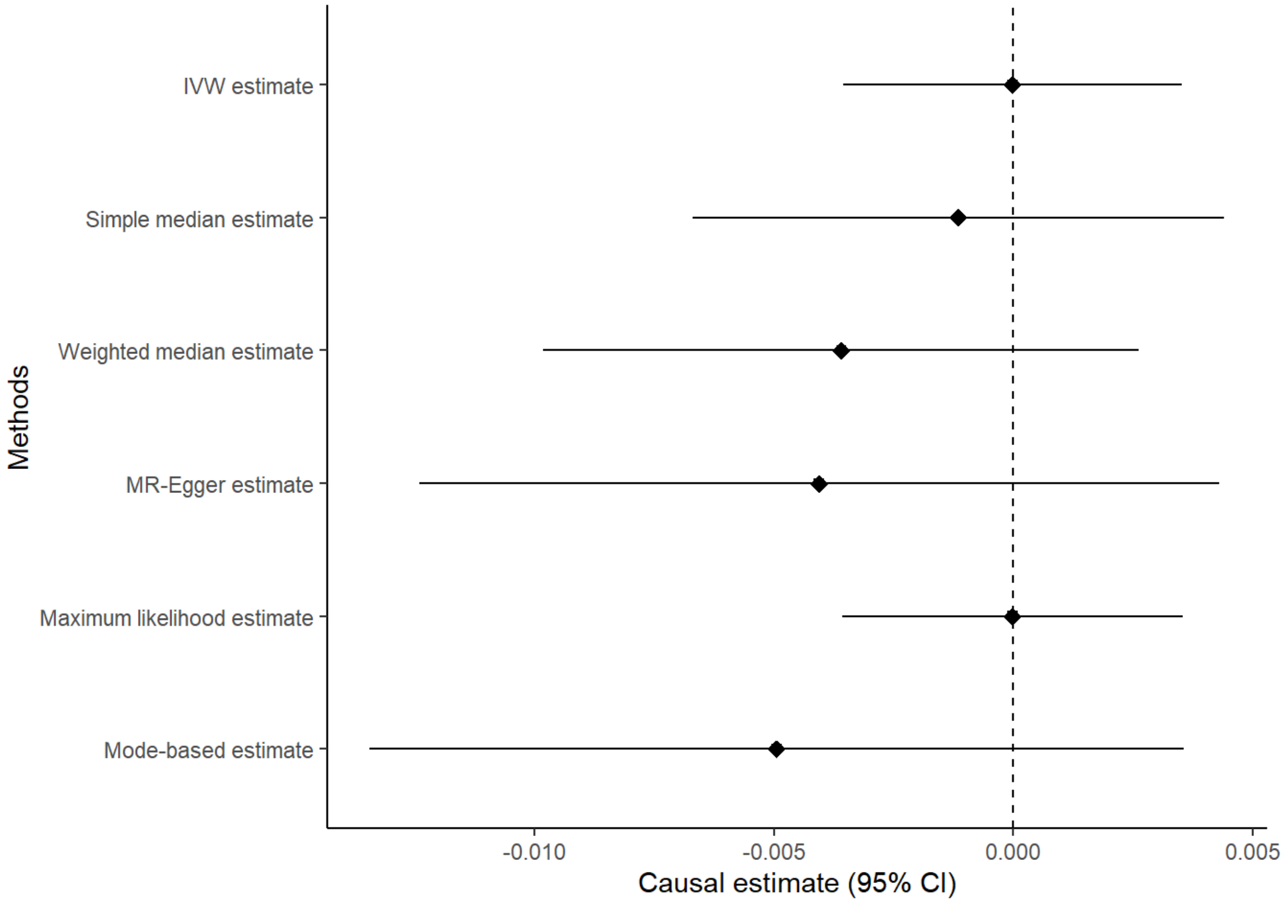
IVW estimate
Simple median estimate
Weighted median estimate
MR-Egger estimate
Maximum likelihood estimate
Mode-based estimate
Methods
-0.010
-0.005
0.000
0.005
Causal estimate (95% CI)

Working with MRraps

```
## $beta.hat
## [1] -1.800092e-05
##
## $beta.se
## [1] 0.001832909
##
## $beta.p.value
## [1] 0.9921641
##
## $naive.se
## [1] 0.001820819
##
## $chi.sq.test
## [1] 144.5566
```

```
##   over.dispersion loss.function      beta.hat     beta.se
## 1           FALSE            l2 -1.800092e-05 0.001832909
## 2           FALSE         huber -1.493614e-04 0.001880525
## 3           FALSE         tukey -1.585695e-04 0.001880529
## 4            TRUE            l2 -1.822860e-05 0.001835206
## 5            TRUE         huber -1.519955e-04 0.001880525
## 6            TRUE         tukey -1.569359e-04 0.001880529
```

```
##
## Constrained maximum likelihood method (MRcML)
## Number of Variants:  172
## Results for:  cML-MA-BIC
## ------------------------------------------------------------------
##      Method Estimate    SE Pvalue         95% CI
##  cML-MA-BIC    0.000 0.002  0.993 [-0.004,0.004]
## ------------------------------------------------------------------
```

```
##
## Debiased inverse-variance weighted method
## (Over.dispersion:TRUE)
##
## Number of Variants : 172
## ------------------------------------------------------------------
##  Method Estimate Std Error  95% CI       p-value Condition
##    dIVW    0.000     0.002 -0.004, 0.004   0.992   992.580
## ------------------------------------------------------------------
```

```
##
## Mode-based method of Hartwig et al
## (weighted, delta standard errors [not assuming NOME], bandwidth factor = 1)
##
## Number of Variants : 172
## ------------------------------------------------------------------
##  Method Estimate Std Error  95% CI       p-value
##     MBE   -0.005     0.004 -0.014, 0.004   0.261
## ------------------------------------------------------------------
```

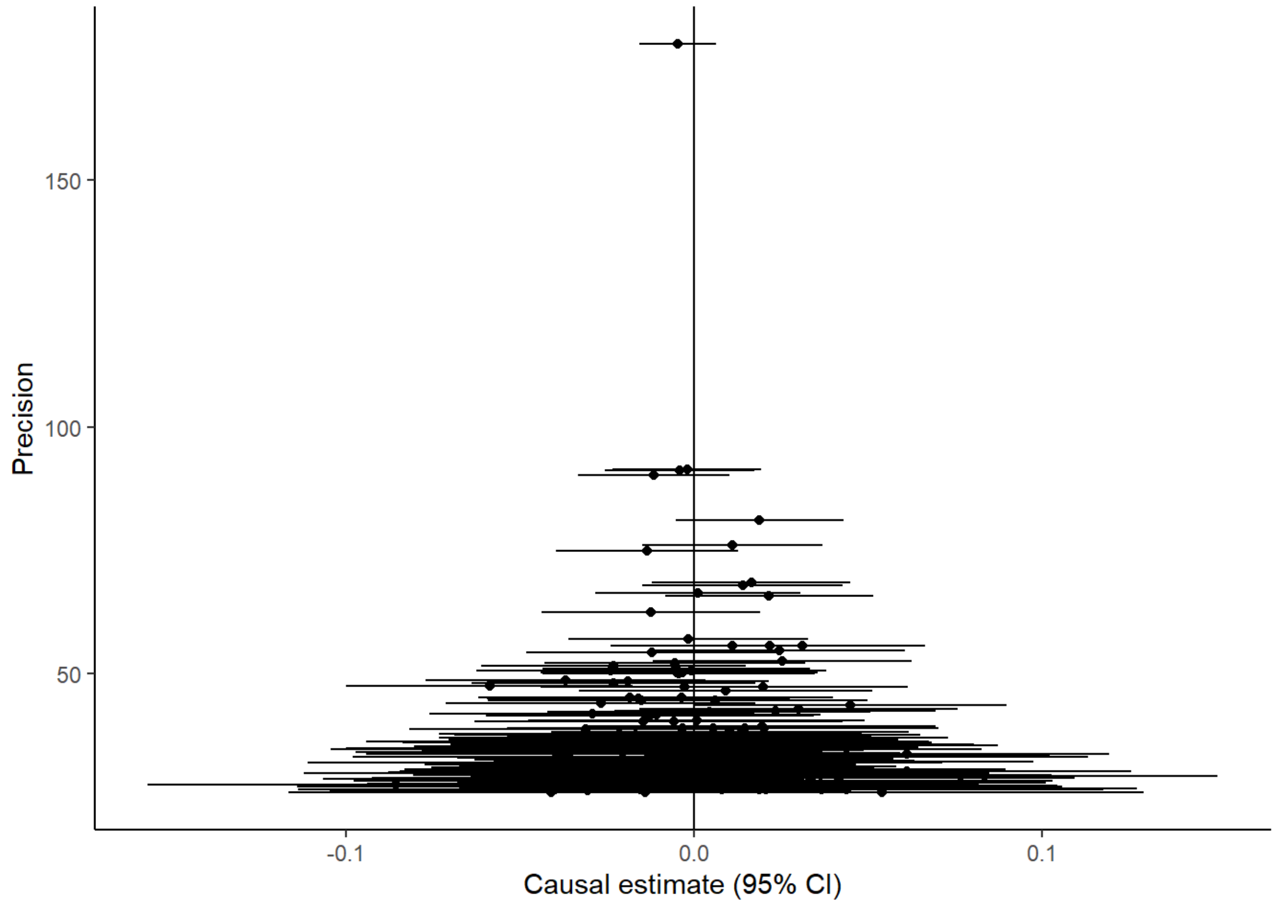
Precision
150
100
50
-0.1
0.0
0.1
Causal estimate (95% CI)

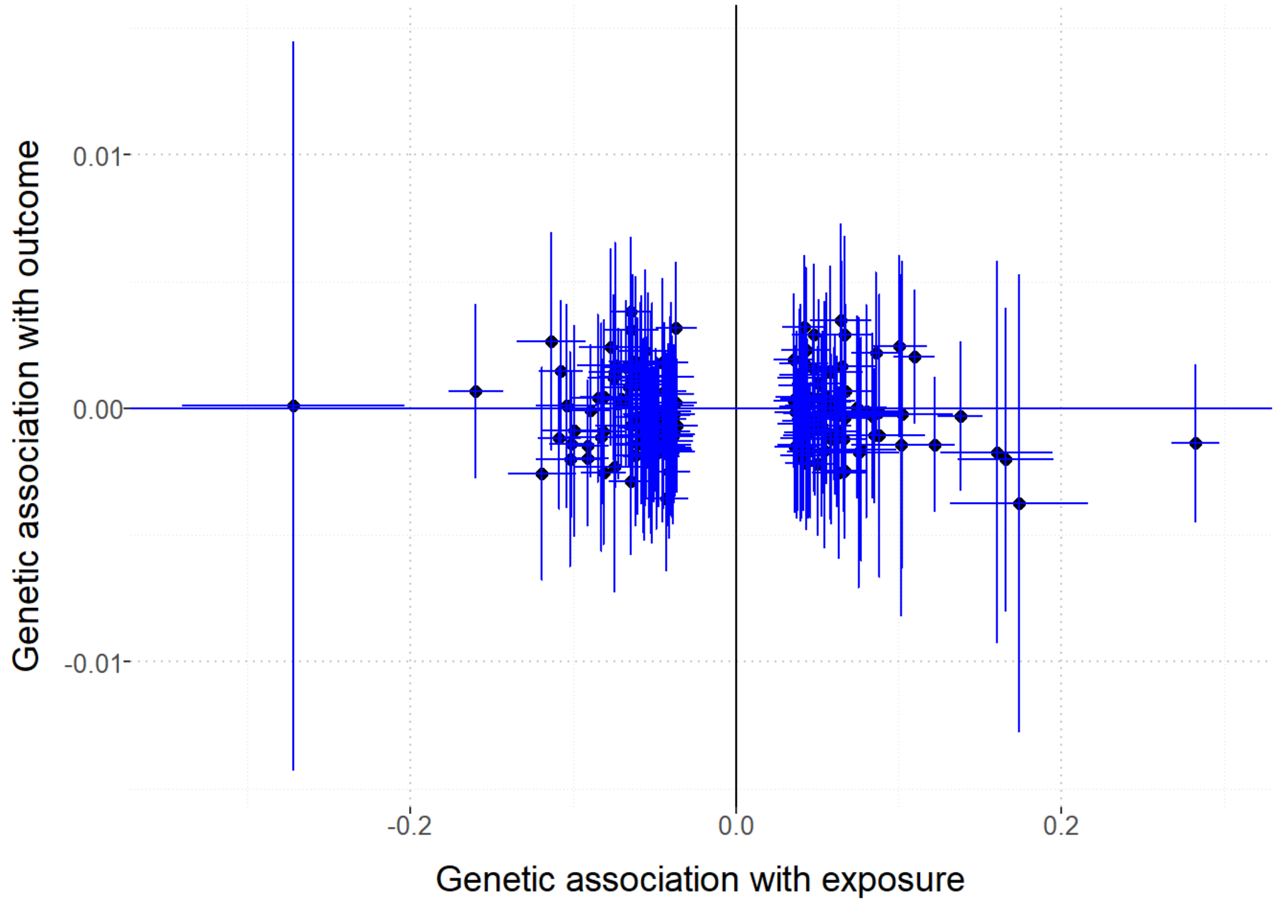
Genetic association with outcome
0.01
0.00
-0.01
-0.2
0.0
0.2
Genetic association with exposure
